# Characterization of Anorexia Nervosa on Social Media: Textual, Visual, Relational, Behavioral, and Demographical Analysis

**DOI:** 10.2196/25925

**Published:** 2021-07-20

**Authors:** Diana Ramírez-Cifuentes, Ana Freire, Ricardo Baeza-Yates, Nadia Sanz Lamora, Aida Álvarez, Alexandre González-Rodríguez, Meritxell Lozano Rochel, Roger Llobet Vives, Diego Alejandro Velazquez, Josep Maria Gonfaus, Jordi Gonzàlez

**Affiliations:** 1 Department of Information and Communication Technologies Universitat Pompeu Fabra Barcelona Spain; 2 UPF Barcelona School of Management Barcelona Spain; 3 Institute for Experiential AI Northeastern University Boston, MA United States; 4 Department of Mental Health Centro de Investigación Biomédica en Red de Salud Mental Parc Tauli University Hospital Sabadell Spain; 5 Parc Tauli Research and Innovation Institute Sabadell Spain; 6 Autonomous University of Barcelona Bellaterra Spain; 7 Fundación Instituto de Trastornos Alimentarios Barcelona Spain; 8 Computer Vision Center Universitat Autonoma de Barcelona Bellaterra Spain; 9 Visual Tagging Services Bellaterra Spain

**Keywords:** social media, Twitter, Spanish, anorexia nervosa, eating disorders, user characterization

## Abstract

**Background:**

Eating disorders are psychological conditions characterized by unhealthy eating habits. Anorexia nervosa (AN) is defined as the belief of being overweight despite being dangerously underweight. The psychological signs involve emotional and behavioral issues. There is evidence that signs and symptoms can manifest on social media, wherein both harmful and beneficial content is shared daily.

**Objective:**

This study aims to characterize Spanish-speaking users showing anorexia signs on Twitter through the extraction and inference of behavioral, demographical, relational, and multimodal data. By using the transtheoretical model of health behavior change, we focus on characterizing and comparing users at the different stages of the model for overcoming AN, including treatment and full recovery periods.

**Methods:**

We analyzed the writings, posting patterns, social relationships, and images shared by Twitter users who underwent different stages of anorexia nervosa and compared the differences among users going through each stage of the illness and users in the control group (ie, users without AN). We also analyzed the topics of interest of their followees (ie, users followed by study participants). We used a clustering approach to distinguish users at an early phase of the illness (precontemplation) from those that recognize that their behavior is problematic (contemplation) and generated models for the detection of tweets and images related to AN. We considered two types of control users—focused control users, which are those that use terms related to anorexia, and random control users.

**Results:**

We found significant differences between users at each stage of the recovery process (*P*<.001) and control groups. Users with AN tweeted more frequently at night, with a median sleep time tweets ratio (STTR) of 0.05, than random control users (STTR=0.04) and focused control users (STTR=0.03). Pictures were relevant for the characterization of users. Focused and random control users were characterized by the use of text in their profile pictures. We also found a strong polarization between focused control users and users in the first stages of the disorder. There was a strong correlation among the shared interests between users with AN and their followees (*ρ*=0.96). In addition, the interests of recovered users and users in treatment were more highly correlated to those corresponding to the focused control group (*ρ*=0.87 for both) than those of AN users (*ρ*=0.67), suggesting a shift in users’ interest during the recovery process.

**Conclusions:**

We mapped the signs of AN to social media context. These results support the findings of previous studies that focused on other languages and involved a deep analysis of the topics of interest of users at each phase of the disorder. The features and patterns identified provide a basis for the development of detection tools and recommender systems.

## Introduction

### Background

Mental disorders are psychological syndromes or patterns associated with distress or disability. Anorexia nervosa (AN) is an eating disorder (ED) characterized by the maintenance of a very low body weight, with a constant desire for thinness and a misconception about body shape [1]. EDs, such as AN, are related to risk factors including perfectionistic traits [2], and the existence of comorbid mood disorders such as depression, of which 33%-50% of anorexia patients experience. Anxiety disorders are common in 50% of these patients [3].

The course of AN is highly variable; however, early intervention is strongly associated with full recovery. The phases that patients go through as they overcome AN can be mapped to the transtheoretical model of health behavior change [4], which is described as an integrative method for understanding how people progress toward adopting and maintaining healthy behaviors. This model identifies the following six stages of change: (1) *precontemplation*, where the individual does not know that there is a problem or that a change is required in their life and, thus, does not seek help; (2) *contemplation*, where the person simultaneously considers and rejects the change, while being conscious of the existence of a problem; (3) *preparation,* where the individual starts to take small steps toward behavioral change, believing that it can lead to a healthier life; (4) *action*, in which the person has changed their behavior and intends to sustain it; (5) *maintenance*, a stage wherein the person has maintained the behavioral change for a considerable period (>6 months); and (6) *termination*, a stage wherein the individual has no desire to return to their unhealthy behaviors. It is important to state that relapse, which implies returning from the *action* or *maintenance* stages to an earlier stage, is likely. This approach has been previously evaluated in patients, confirming that the notion of the stages of change is an independent dimension that is very relevant for the treatment of EDs [5].

Automated methods have been designed to detect signs of AN, some of which address the development of early detection approaches [6,7], as it has been proven that the signs and symptoms of mental disorders, including AN, can be traced using social media [6,8-13]. The findings of such research have revealed patterns that can be relevant for the development of tools to detect harmful content [6] and to assist clinicians and psychologists in screening [10-12] and treatment proceedings [6]. Research findings on these topics can also contribute to the improvement of the structure and services provided by online social platforms [6], which are a means through which people with mental disorders can find support for their recovery, as well as they can be used as tools to promote harmful content, which is the case for suicide promoters and pro–eating disorder (pro-ED) communities [6,13].

Recent approaches for characterizing AN on social media account for individual posts from users, meaning that the signs and symptoms are traced by analyzing the content of a single post per user (post level) [6,8,9]. In contrast, there are only a few approaches that address multiple posts of a single user (user level) [10,11]. There are no approaches that address the simultaneous analysis of relational, behavioral, demographical, and multimodal information at the user level, although few studies have combined some of these aspects on AN-related disorders such as anxiety, depression, and suicidal ideation [12,13].

With regard to the type of information extracted and analyzed about EDs on social media, several state-of-the-art approaches are dedicated to the analysis of textual information contained in posts [6,9,14]. They analyze the topics of interest using topic modeling techniques [11], and they also consider the frequency of the terms used through bag-of-words (BoW) models and n-grams [7]. Other textual elements are also considered, such as the frequency of terms closely related to the illness and other lexical and syntactic elements [11,14]. In addition, sentiment analysis tools [15] and methods that use word embeddings, which are vector representations of terms, have been applied [7,16]. Most of these features are later used as representations suitable for the development of predictive models that are often based on machine learning methods [6,7,16]. Through the analysis of text cues, it has been found that users with depression and those with suicidal ideation make more self-references [13], whereas proanorexia and prorecovery communities exhibit distinctive affective, social, cognitive, and linguistic style markers, as proanorexics express greater negative affect, feelings of social isolation, and self-harm [6].

With regard to the analysis of relational information, Wang et al [17,18] analyzed the interactions between users with EDs to identify the topics shared in web-based conversations. In particular, they explored the interactions among communities with different stances on EDs, considering both pro-ED and prorecovery content, and reported that intercommunity interactions are very limited. In the context of similar disorders, De Choudhury et al [19] constructed egocentric social graphs for postpartum depression detection and found that increased social isolation and reduced availability of social capital on Facebook are the best predictors of postpartum depression detection among mothers. In addition, Colombo et al [20] studied the connectivity and communication of suicidal users on Twitter based on the evaluation of retweets.

Regarding the analysis of images shared by users, only a few studies analyzing the content of images to determine patterns that can be linked to AN signs are available in the literature. This aspect of analysis is important for studies on EDs, as the symptoms of AN, such as extreme weight loss, an obsessive interest in excessive exercise, and restricted food intake through dieting, can be identified from images [1]. Chancellor et al [21] focused on Instagram, which mainly includes image posts, but they exclusively studied textual (captions) and relational elements. Recently, only a few approaches have addressed the actual analysis of image properties to detect mental disorders, such as self-harm, depression, and anxiety [12,22,23]. To the extent of our knowledge, the work of Chancellor et al [16] is the only available study on EDs that analyzed images. Chancellor et al [16] classified moderated pro-ED content and found that the identification of deviant pro-ED content through the analysis of images was possible, reaching a recall of 84%. This approach mainly focused on the detection of harmful images but did not include the analysis of interpretable elements and patterns that might characterize AN-related content on images.

There are also a few studies that consider the stages that patients go through during the course of the illness. De Choudhury [6] and Wang et al [17] addressed the detection and characterization of prorecovery communities, which can be distinguished from pro-ED communities. However, both of these communities did not exclusively include people with the illness. Chancellor et al [24] determined the content and participation measures that could predict the likelihood of recovery of these types of users on Tumblr. They found that only half of the analyzed users were likely to exhibit signs of recovery after a 4-year period, demonstrating the importance of further consideration of the stages that lead to recovery, thus supporting the *transtheoretical model*.

Regarding the analysis of content generated by Spanish speakers with EDs, we identified two approaches that address writings in this language [9,25]. Both approaches are focused on the analysis and detection of users with AN at the post level, with a main focus on textual-based features. Furthermore, these approaches do not address any further recovery stages. Recent works have also addressed other mental conditions such as depression [26] and suicidal ideation [13].

### Objectives

This paper addresses the characterization of EDs, in particular AN, in social media. We aim to answer the following research questions (RQs): (1) Which are the visual, textual, behavioral, and demographical elements that characterize and distinguish users at the early, treatment, and full recovery stages of AN on social platforms?; (2) Which elements characterize the social network of users that use anorexia-related terms?; (3) Which are the topics of interest among AN users and their followees?; (4) Which linguistic attributes characterize Spanish-speaking users with AN?; (5) Does the proportion of tweets related to AN significantly change according to the recovery stage?; (6) In a social platform context, can the elements that distinguish users at the precontemplation stage from users at the contemplation stage be identified?; and (7) Are there significant differences between the focused and random control groups?

The main contributions of this work are as follows:

- We generated a Twitter data set that is annotated by psychologists, psychiatrists, and therapists and consists of writings of users manifesting AN (including writings corresponding to treatment and of fully recovered users), and two types of control cases (ie, focused and random control). To the best of our knowledge, this is the first Spanish data set for the analysis of AN at the user level that considers different stages of the illness toward recovery.
- We extracted and inferred several features that consider multiple elements, as follows: images, texts, relations among users, posting patterns, and demographic information. These features were generated to identify elements that characterize users with AN at different stages of the illness and recovery. We also determined the elements that distinguish these users from the two types of control cases.
- We established a deep learning–based method to identify tweets related to AN using individual tweets.
- We trained a predictive model using the images of users with AN and control users to detect whether differences between these groups can be identified on the basis of visual properties.
- We further explored the social network of users with AN through the detection of communities and the analysis of topics of interest of the different types of users, along with those of their followees.
- We applied a clustering approach to distinguish users at the *precontemplation* stage from those at the *contemplation* stage using the *transtheoretical model*.

## Methods

### Data Collection

We chose Twitter as our main data source, as it has been previously used for studying mental disorders, including EDs, on social media [14,27]. Twitter is suitable as it allows the collection of a set of chronologically organized posts in Spanish and provides metadata relevant for analyzing the relational and behavioral aspects of users.

We selected keywords and popular hashtags commonly used by ED communities, phrases likely to be used by people undergoing treatment, and terms used by recovered users. These keywords and phrases were manually collected and classified from multiple sources in Spanish and English, including proana blogs, academic publications, and documents made available by the Spanish Association Against Anorexia and Bulimia [14,28,29].

In addition, we conducted a survey among volunteers who have recovered from AN. The phrases and keywords collected were evaluated and filtered by clinicians that were asked to agree on choosing up to 30 keywords or phrases in Spanish that would lead to reach posts from users with AN, including *proana, peso objetivo (objective weight), perder peso (lose weight), IMC (body mass index BMI), sibutramina (sibutramine), mi anorexia (my anorexia), ana y mia (ana and mia)*. We collected 114,627 public tweets containing the search phrases, considering a period in the past, from December 21, 2017, to December 21, 2018. At the same time, a sample of up to 10,000 tweets from the same search period was collected for each user.

To protect the privacy and identity of these users, generic identifiers were assigned to each of them and their posts. In addition, we removed any personal information from the users’ descriptions and tweet texts such as usernames, names (proper nouns), email addresses, URLs, password combinations, location names, and all numbers. The extracted metadata elements and texts passed through a strict transformation process to build and exclusively store vector representations of the features of interest, guaranteeing the analysis of fully anonymized information.

### Annotation Process

For labeling purposes, we filtered and only considered users with at least three different tweets containing the selected keywords for each category. Among all categories, 645 users met this criterion. Before the submission of the text samples to the annotators, the sample of tweets' texts selected for annotation were anonymized and translated to English to avoid the reidentification of users based on their writings.

We defined five independent groups of users: (1) AN users that manifest the first stages of the disorder and describe signs and symptoms of AN in their texts, which includes users at both the precontemplation and contemplation stages according to the *transtheoretical model*; (2) a focused control group in which, similar to our prior work [13], we included users that did not manifest signs of anorexia but use terms related to the disorder in their writings; (3) treatment users that explicitly stated that they have been diagnosed with AN and are in treatment; (4) recovered users who claim they have recovered from AN; and (5) doubtful cases in which clinicians were not sure about any of the prior categories. A total of 5 annotators participated in the labeling process: 3 psychologists and 2 psychiatrists. These annotators collaborated closely with organizations specializing in the treatment of EDs. The final label for a user’s set of tweets was assigned if at least three annotators agreed on the assigned label. For cases where an agreement was not met, the users’ tweets were categorized as doubtful cases.

From this first classification approach, a total of 195 users were classified as users with AN, 283 as focused control users, 29 as under treatment users, 18 as recovered users, and 119 as doubtful cases. We performed an interannotator agreement analysis and obtained a Light κ coefficient of 0.4751 (*P*<.001), which is the result of the averaged Cohen κ values calculated between each pair of annotators. This approach was chosen over Fleiss κ, as all annotators evaluated every sample. The values obtained suggest a moderate agreement among annotators.

In addition to the focused control group, we included another control group consisting of 223 randomly selected users called *random control users*. These users did not necessarily use terms related to AN and were selected using Twitter’s Sample Tweets application programming interface (API) [30], which provides a set of random tweets, from which we could obtain the users for our sample. By analyzing the description and a sample of their tweets, we ensured that no users with AN were part of this group. The anonymization and translation process applied for the other groups was followed for this case. Table 1 summarizes the user groups defined initially for the study.

**Table 1:** User groups defined for the study (N=748).

Group	Description	Collection method	Users, n (%)
AN^a^	Users most likely at early and advanced stages of AN that do not seem to be on treatment This group corresponds to the precontemplation and contemplation phases of the transtheoretical model.	AN-related keywords using the Twitter Search API^b^	195 (26.1)
Treatment	Users most likely in treatment of AN This group corresponds to the preparation, action, and maintenance phases of the transtheoretical model.	AN- and treatment-related keywords using the Twitter Search API	29 (3.9)
Recovered	Users that seem to be recovered from AN and have reached the termination stage They should specify not having a relapse in a long period (≥4 years).	AN- and recovery-related keywords using the Twitter Search API	18 (2.4)
Focused control	Control users that use vocabulary related to AN, such as psychologists, news accounts, or medical centers.	AN-related keywords using the Twitter Search API	283 (37.8)
Random control	Twitter users randomly selected with no signs or symptoms of AN	Sample tweets using the Twitter Search API	223 (29.8)

^a^AN: anorexia nervosa.

^b^API: application programming interface.

### Characterizing Users With AN on Social Media

This work is dedicated to the characterization of AN considering the multiple stages through which people on the recovery path go through (RQ1), including the analysis of the differences between the two control groups analyzed: focused and random control (RQ7). Our goal is to identify the elements and patterns that distinguish people at each phase based on the analysis of multiple perspectives that are usually considered by clinicians for screening and treatment purposes. The perspectives analyzed include (1) the way Spanish-speaking users express themselves through written posts (content shared) that can lead to the exploration of linguistic dimensions, affective processes and emotions, personal concerns, topics of interest, vocabulary that implies the existence of risk factors associated with the illness, and vocabulary related to signs and symptoms of AN (RQ4); (2) the social network of users, with a focus on the interests shared with their followees and the detection of communities (RQ2 and RQ3); (3) the users’ behavior, with the analysis of posting patterns in different periods; (4) demographic aspects such as gender and age ranges; and (5) visual elements that include the analysis of the characteristics of the profile picture of users, along with the images shared on posts.

In addition to the analysis of prior perspectives, for a further exploration of textual cues, we built and evaluated classifiers for the detection of individual tweets related to AN. These tools can be useful for the detection of users with AN, as we assume that a larger number of tweets related to anorexia will be found in the profiles of users living with the disorder. Through this analysis, we also wanted to analyze whether the proportion of tweets related to AN changes significantly according to the recovery stage (RQ5). In addition, these tools can later be useful for automatically filtering writings that are exclusively related to the condition. They can also be used for screening and annotation purposes [13].

We also tested a clustering approach to distinguish cases at the precontemplation stage from cases at the contemplation stage (RQ6). People in the precontemplation phase (also known as the *honeymoon* phase) are characterized by being enthusiastic about their weight loss and the social support they receive. This is a phase in which people believe they are in control of their behavior, and therefore, they are in denial of their unhealthy condition. This behavior differs from the contemplation stage, in which bad habits and symptoms remain, but as the illness progresses, the person notices that there is something wrong and feels that they are losing control of the situation. People at this stage are constantly depressed and may even experience a decrease in their cognitive capabilities [31]. Taking into account these aspects, we consider the detection task relevant, as people at the contemplation stage are more willing to seek help, and therefore, a potential nonintrusive intervention through social media, such as social recommendation, could be successful having users at this stage as a target. However, such potential applications must be further analyzed by considering all the privacy and ethical aspects involved.

### Experimental Setup

#### Data Set Description

The data set built for our analysis consists of a set of features generated and inferred based on the text, images, and metadata of the annotated users’ tweets. Table 2 provides relevant information regarding each group in our data collection. For each user, we considered the content from their profiles (tweets) during a year (from December 21, 2017, to December 21, 2018). Some of the initially labeled users were not further considered in the data set, as they had published less than five tweets during the data collection period, which we considered as not informative enough for our analysis purposes. A total of 694 users were part of our final data set, which contained data collected from 2,133,110 tweets, including 405,909 images. It is important to recall that retweets were not considered within the scope of our analysis.

**Table 2:** Data set description.

Descriptive item	User group				
	Anorexia nervosa	Treatment	Recovered	Focused control	Random control
Users (n=694), n (%)	171 (24.6)	27 (3.9)	18 (2.6)	271 (39)	207 (29.8)
Tweets collected (n=2,133,110), n (%)	434,615 (20.4)	8317 (0.4)	52,578 (2.5)	1,109,861 (52)	447,739 (21)
Number of tweets collected per user (n=2,133,110), median	1239	1748	2036.5	2608	873
Tweet length (number of words; n=2,133,110), median	14.00	14.00	13.50	12.50	19.00
Images (n=405,909), n (%)	40,142 (9.9)	6584 (1.6)	4202 (1)	298,488 (73.5)	56,493 (13.9)

#### Comparative Analysis and Evaluation Measures

As part of our data collection process, we extracted, calculated, and inferred some features for performing the analyses required to answer our RQs. For this purpose, we considered network clustering and visualization algorithms (RQ2 and RQ3); prebuilt machine learning models for sentiment analysis; and age range and gender detection tools, including models for the detection of objects in images (RQ1, RQ4, RQ5, RQ6, and RQ7). We also used external sources with lexicons to detect emotions, topics of interest, risk factors, and affective processes lexicons (RQ1, RQ4, RQ6, and RQ7). The description of the extracted features and the calculation or inference processes are detailed in the *Analyzed Perspectives and Features* section. Phyton (Python Software Foundation) was used as our main programming language for data extraction and processing procedures.

To perform a comparison of the groups analyzed, we considered some approaches for hypothesis testing. To do so, we first verified that our numerical features did not follow a normal distribution and that there was no homogeneity of variance for most of them. Therefore, we considered nonparametric tests. First, we applied a Kruskal-Wallis test [32] for multiple independent samples, and after finding features with significant differences among the groups, we proceeded to perform the Mann-Whitney *U* [33] test to check for differences between pairs of groups of interest. As we considered some categorical elements as well, such as age groups, we transformed them into Boolean representations to perform a two-sided proportion *z* test among the groups with these feature types, which is a test equivalent to the proportions chi-square test [34].

Our work addressed three predictive tasks solved using machine learning: one to build a classifier that identifies tweets related to AN (RQ5), another to distinguish users in the precontemplation phase from users in the contemplation phase (RQ6), and the last one to detect individual images related to AN (RQ1). We generated deep learning models based on convolutional neural networks (CNNs) [35] and statistical models such as logistic regression (LR) [36] for the detection of texts and images related to anorexia, taking into account these as supervised tasks for which we have annotated data to learn from. Later, we consider a clustering approach given by a k-means classifier for the detection of users at the precontemplation and contemplation stages, as we did not have data annotated for this purpose. Our predictive approaches were evaluated using machine learning evaluation measures, such as precision, recall, and F1 score. The details of these approaches are described in the *Proportion of AN-Related Tweets, Detection of Precontemplation and Contemplation Phases,* and *Pictures Shared* sections.

#### Analyzed Perspectives and Features

##### Overview

In this section, we describe the perspectives and specific features analyzed for the characterization of AN across its stages. The perspectives and categories of the features considered within each perspective are studied by considering their relation to elements that clinicians address for screening and treatment purposes [1,2] and their use for the characterization of related mental health issues such as anxiety [12], depression [23,26], and suicidal ideation [13,20].

##### Content Shared and Interests

###### Overview

This perspective addresses RQ1, RQ3, RQ4, RQ5, and RQ7 through an analysis of the textual content shared by users in their tweets. It considers linguistic and psychological aspects through six categories. Some of these categories were based on a classification given by the Linguistic Inquiry and Word Count (LIWC) 2007 Spanish dictionary [37,38], which categorizes words into psychologically meaningful categories. The remaining categories were defined by considering psychological aspects related to EDs, which were defined under the supervision of clinicians. The categories analyzed were as follows: linguistic dimensions (24 features) [37,38], affective processes and emotions (29 features) [37,38], personal concerns and biological processes (12 features) [37,38], vocabulary related to risk factors (10 features) [1,2,13], anorexia-related vocabulary (9 features) [14], and topics of interest to the users (200 topics). From this perspective, we aim to explore the linguistic factors and shared interests that characterize each group. In addition, we map certain elements that are relevant for specialists when identifying signs and symptoms of AN, such as the existence of risk factors, the use of terms related to the illness, and the expression of emotions and personal concerns of the users.

###### Linguistic Dimensions

These features were extracted using a LIWC dictionary [37,38]. The features belonging to this category address the use of grammatical and syntactical elements such as pronouns, verbs, adverbs, prepositions, and articles, taking into account the different verbal times and pronoun types. These are relevant elements for our analysis because they can give us insight into the elements or people that users are drawing their attention to. These types of elements have been analyzed in prior work where other mental disorders such as depression are analyzed; for instance, Rude et al [39] in their work on depression found that people who are experiencing physical or emotional pain tend to draw their attention to themselves and therefore use more first-person singular pronouns. For our approach, after concatenating all the tweets’ texts of a user into one long text, we counted the frequency of words belonging to each of the categories considered from the dictionary, which was normalized by the size (in number of terms) of the resulting concatenation. In addition to the LIWC elements, we also considered the median tweet length (number of terms) as a feature. With the analysis of these features, we also check for the elements that characterize the linguistic attributes of Spanish speakers living with this disorder so that they can be compared with prior studies in English (RQ4).

###### Affective Processes and Emotions

These features are extracted using the LIWC dictionary and EmoLex [40], which is a dictionary that associates words with eight basic emotions, anger, fear, anticipation, trust, surprise, sadness, joy, and disgust, and two sentiments, negative and positive. This dictionary also provides a file to associate the original terms in English with their Spanish translations. Along with the emotions explored, we addressed cognitive processes, senses, perceptions, and social processes (LIWC). The values obtained for a given user were calculated in the same way as the values for the linguistic dimensions. In fact, the same calculation approach is also used for *personal concerns*, *risk factors*, and *anorexia-related vocabulary* features. The analysis of this aspect is relevant, as general psychiatric disturbance and negative emotionality are elements that characterize people living with EDs [2].

In addition to the prior elements, we make use of a sentiment analysis model, which provides a polarity value for an individual text, in the range of [0,1], from the most negative to the most positive polarity. We used Senti-py [41], trained on Spanish texts from different sources, including Twitter. It is based on a BoW model that goes through an intermediate feature selection process. To obtain a score per user, we calculated the median polarity scores of all the tweets. The higher the polarity score obtained, the more positive the text was expected.

###### Personal Concerns and Biological Processes

This is another perspective addressed through the analysis of lexicons (LIWC). The elements explored provide a general perspective of the use of terms related to aspects that involve daily activities and concerns, along with biological aspects. Within these aspects, we find terms related to religion; work; leisure; money; and biological processes, such as body, ingestion, and health. These aspects are relevant to our study, as personal background aspects are considered to be relevant for the development of EDs [2]. Biological processes are also relevant to address, as these concerns are representative of patients with anorexia [1]. It is important to specify that we only address the use of general terms such as *God*, *doctor*, or *office*, for instance, we do not do a further exploration regarding personal religious beliefs, professions, or health information within the text.

###### Vocabulary of Risk Factors

These features correspond to lexicons consisting of up to 3-grams phrases that were mapped to ED risk factors, such as terms referring to self-injuries; suicidal ideation references; self-loathing terms; words that express disdain, substance abuse, lack of social support, family issues; and vocabulary, which refers to discrimination- or abuse-related topics [2,13,42]. The terms and phrases selected for these categories were based on manually mapping common terms and phrases related to anorexia and the terms used in a related task for the detection of suicidal ideation [13].

###### Anorexia-Related Vocabulary

On the basis of the work of Arseniev et al [14], we used the categories of terms related to AN and its symptoms. Subsequently, we translated them into Spanish. We also kept some of the terms in English, as they are also used by Spanish-speaking users. In addition to these categories, we added names of known laxatives in Spanish [43]. These categories refer to topics such as anorexia promotion, body image, body weight, food and meals, caloric restrictions, compensatory behaviors, and exercise.

###### Topics of Interest

The topics of interest of a user are also among the elements that we analyze from the content perspective (RQ3), as we would like to know if there is a shift in the main interests of users through the recovery path. For this purpose, we consider predefined topics of interest based on categories that are part of the Empath tool [44], which generates and validates lexical categories on demand from a small set of seed terms (such as football and tennis to generate a sports category). This tool draws connotations between words and phrases by deep learning a neural embedding using a corpus with 1.8 billion words. In our case, 200 prebuilt topics are considered, including sports, music, social media, and politics, among others.

For each user, we calculated the scores for all topics. We take into account that the topics of interest of a user are given by the interests of their followees, the content they like (given by the tweets made by others and marked as favorites), and by the content posted by themselves. For each user, we collected (1) a random sample of their own tweets (up to 500 texts), (2) a random sample of 200 tweets that they had liked during the same period, and (3) the profile descriptions (biographies) of up to 200 random followees of the user. These tweets and descriptions were relevant enough samples of texts that characterized the interests of a user. An individual score with Empath was obtained for each text (tweet or description). Later, the final score for a topic for a given user was calculated by averaging the scores obtained by the topic on all the tweets considered. It is important to mention that as Empath’s categories are in English, we add a translation step before the Empath scores’ calculation, using the *Googletrans* Python API [45] for this purpose. The amounts of tweets and descriptions defined for this approach are also based on the request limitations of the API of Twitter and the Googletrans API*.*

###### Proportion of AN-Related Tweets

Our RQ5 analyzes whether the proportion of tweets related to AN changes significantly according to the recovery stage, as it is expected that users at the initial stages produce more tweets related to their condition. For this purpose, we first built and compared two models to detect, for each user, if each of their tweets are related to AN. Second, we calculated the median score obtained by the classifier for all user tweets. Finally, we compared the median values for all users belonging to a group to measure the presence of AN tweets in each group. It is expected that users with AN have a median value significantly higher than users in the control and recovered groups. The approach of creating classifiers such as these has previously been used to detect users with suicidal ideations [13]. In this case, the model was built exclusively using a BoW model and LR.

For the AN use case, we trained two classifiers to distinguish tweets of two classes: (1) *anorexia related* and (2) *control.* The instances of the anorexia-related class corresponded to the individual tweets belonging to the users labeled as AN cases (1766 tweets). Later, an equivalent number of tweets was selected to represent the control class; these tweets were randomly extracted using Twitter’s Sample Tweets API [30]. The sample of tweets collected was reviewed by annotators to discard those that could possibly belong to the anorexia-related class.

The first classifier was trained over a BoW model with (1-3)-grams at a term level. For this purpose, we used the *Scikit-learn* [46] Python library: *TfIdfVectorizer* to generate a *tf.idf* representation with (1-3)-grams. We considered Spanish stop words [47] and used *ekphrasis* [48] as a text preprocessing tool to replace terms referring to money, hashtags, and emoticons with generic tags. As we obtained 66,404 features, we reduced the number of features to 300 using principal component analysis. We used an LR method and 10-fold cross validation.

For the second classifier, a deep learning approach was applied. The model was defined through a CNN architecture that has been previously applied to text classification tasks [35], including a similar task for suicide risk assessment on social media [13,49]. The same preprocessing approach as that used for the prior model was applied. To train this model, tweets were represented as sequences of terms, and these terms were represented by prelearned word embeddings that were trained over tweets in Spanish [50]. Each tweet was considered as an instance, and its label (anorexia related or control) corresponded to the class assigned to the tweet. For the CNN [35], the embedding sequence instances were given as the model input, where a task-oriented fine-tuning was performed, and we applied a filter window ({2,3,5} terms). We applied max pooling and passed the output to a sigmoid layer to generate the final output. Furthermore, 75% (2649/3532) of the instances were selected for training purposes and the remaining 25% (883/3532) for testing. Among the training instances (tweets), 69.98% (1854/2649) were selected for training the model and 30.01% (795/2649) were considered for validation.

##### Social Network

###### Overview

This perspective addresses RQ1, RQ2, and RQ3. Features are extracted taking into account the social network of the user, as elements related to this perspective have been proved to be useful for characterizing other mental conditions such as depression [19] and suicidal ideation [20]. We analyze some features that characterize the user´s popularity and the support received by other users. These features correspond to the number of followers, favorites, and retweets of their posts. We focused on the social network (followees) of users that make use of anorexia-related terms (RQ2), as our goal was to detect communities among these types of users. Furthermore, we explored the likelihood of users with AN to follow users living with the disorder or anorexia promoters by analyzing the topics of interest of their followees. We also explored the differences in their interests and those of the followees of users in treatment and the followees of recovered users.

###### Measures of Interactions and Engagement

These features are extracted from the metadata of the users’ tweets. These features tell us about the relationships and interactions of AN users, which can differ from the interactions of control users [19]. The features extracted and calculated for each user are as follows: number of followees, number of followers, total number of favorites given to the posts of other users, median number of favorites received by the user, and median number of retweets received by the user. These last two features were calculated by considering the tweets of the sample of the user profile.

###### Analysis of Followees and Community Detection

As part of the perspective that analyzes the social network of a user, we explored the network of users that made use of anorexia-related terms, corresponding to the AN, treatment, recovered, and focused control groups. This was done with the purpose of identifying characteristics of the network that were capable of distinguishing the groups defined, in particular the AN group and the focused control group, as users representing organizations that provide medical and psychological support could be part of it, and it would be relevant to get an insight into the relationships between both groups. For this purpose, we extracted a sample of up to 100 followees of each user from each of these groups (considering Twitter’s API request limitations). We built a directed graph where a link between two nodes was given by a *follows* relationship, meaning that users, represented by nodes, are linked to other nodes through directed edges where the arrowheads point to the users they follow. Later, a clustering algorithm was applied to detect communities among these users. We then performed a comparison between the communities automatically detected and what we defined as validation groups, which were created considering the followees of the AN, treatment, recovered, and focused control groups. These validation groups were defined in such a way that a user was assigned to a validation group (AN, treatment, recovery, or control) if it was mostly followed by users belonging to the originally labeled groups. This was done taking into account up to two followees’ levels, denoted as validation subgroups, as explained in Table 3, where we describe the general organization of a group. We considered four main groups and three subgroups per group, where the first subgroup always corresponded to the original users labeled. An instance of a validation group would be *Group AN,* which is composed of three subgroups: *G_1_* composed of the originally labeled AN’s users, *G_2_* composed of the users mostly followed by *G_1_* users, and *G_3_* composed mostly of users followed by *G_2_*.

**Table 3:** Groups for social network analysis based on users’ labels.

Group and subgroup			Nodes user type
**Group X^a^**			
	Subgroup G^b^_i_	Users manually labeled as part of the X group	
	Subgroup G_i+1_	Users mostly followed by G_i_	
	Subgroup G_i+2_	Users mostly followed by G_i+1_	

^a^X: anorexia nervosa; focused control; treatment or recovered.

^b^G: group.

On the basis of a manual revision of a sample (translated to English) of the profile descriptions of users belonging to the communities detected with most nodes, we performed a further analysis of the types of users that were identified as part of each community, and we mapped these communities to our predefined groups so that we could identify which type of users from our groups of interest were part of the communities detected. For the visualization of the social network, we considered the Force Atlas 2 [51] algorithm, and for the detection of communities, we used the method by Louvain [52], both implemented on Gephi [53].

###### Analysis of Interests Between Users and Their Followees

**Figure 1:**
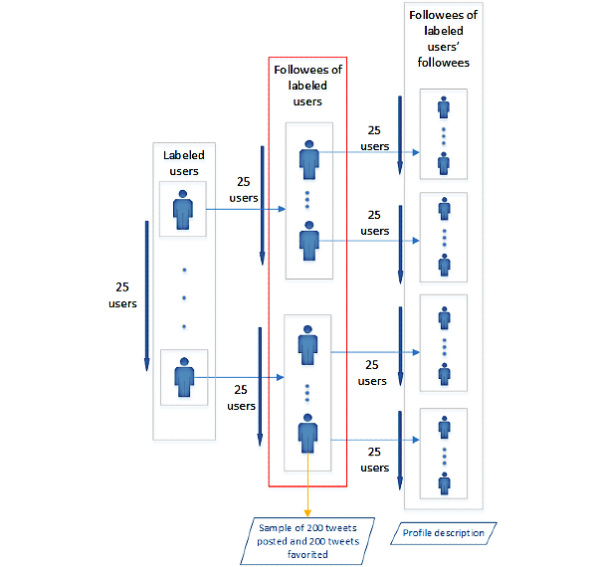
Structure defined for the extraction of the interests of the followees of a given user group. For each labeled user of a group, we analyzed the tweets posted and liked by their followees and the profile description of the followees of the labeled users’ followees.

To address RQ3, as it is our purpose to identify the topics of interest of AN users’ followees, we follow the process applied for the analysis of the topics of interest of users of each group, as described in the *Topics of Interest* section, but in this case, we address the followees of each user type. As shown in Figure 1, for this case, we considered up to 25 followees from a sample of up to 25 users per group analyzed. Then, for each of these followees, we calculated scores for the Empath topics by considering the descriptions of 25 random followees, a random sample of their own tweets (up to 200 texts), and a random sample of 200 tweets that they had liked during the same period. The score of a topic for each followee of a user is given again by the average score obtained from all the texts considered. The score for a topic of a user is given by the median of the scores of their followees. Once the scores for the samples of users representing each group were obtained, we calculated the median value of each topic using the scores of each user belonging to the group. Later, we performed a comparison between the interests of users of each group (calculated before) and those of their followees.

##### Behavioral Aspects

Addressing RQ1 and RQ7 from this perspective, we define features that measure the users’ activity to explore elements that can link the frequency of use of social platforms and AN. An instance is the level of activity of users at night, which might imply insomnia, a sign that has been linked to related disorders such as suicidal ideation [13]. These features were extracted from the metadata of the tweets, and they measured the behavior of users based on their activity on a daily, weekly, and monthly basis. The features considered are as follows: the number of tweets on weekdays (Monday-Friday) normalized by the number of tweets from the whole week (weekly tweets ratio), the number of tweets on weekend days (Saturday and Sunday) normalized by the total number of tweets of the week (weekend tweets ratio), the number of tweets posted by the user within each quarter of the year normalized by the total number of tweets of the year period (year quarter tweets ratio), the total number of tweets posted during the year, the median time (in seconds) that passes between the publication of each tweet, and the ratio of tweets posted during the regular sleep time of the user (sleep period tweeting ratio). We also calculated the ratio of tweets posted during the period when the user was usually awake (awake period tweeting ratio). Both of these last features were calculated based on the *sleep time tweets ratio (STTR)* and the *day time tweets ratio (DTTR)* inference approach [13]. This approach was adopted because it addresses the issue of the lack of information regarding the location of the users, which is mostly not shared by them.

For the calculation of the sleep period tweeting ratio, equivalent to the STTR (equation 1) [13], a day is divided into eight fixed time slots of 3 hours each, and it is assumed that an average user has approximately 6 hours of sleep time. This is precisely the period where a fewer number of tweets would be created compared with the rest of the day, so the number of tweets (t) created within each 3-hour time slot is counted for all the tweets of the user, and the sum of the number of tweets within each pair of continuous time slots is calculated. Then, the minimum score obtained by all the pairs is selected, so that later this value can be normalized according to the total number of tweets of the user (T). It is assumed that the first and last slots are continuous. The awake period tweeting ratio is given by the DTTR, which is equivalent to the difference between 1 and the sleep time ratio: DTTR=1−STTR.




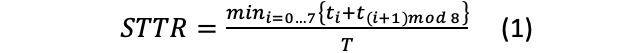




##### Demographics

We analyzed the demographic characteristics (gender and age features) of the groups to verify whether these correspond to the actual incidence rates [54] (RQ1 and RQ7). These features are inferred, given the fact that Twitter does not publicly display the age and gender of users. We used the approach of Wang et al [55] for demographic inferences. This approach is based on a multimodal deep neural architecture for the joint classification of age, gender, and organizational status of social media users. Their model was trained using data in 32 languages, including Spanish. The method analyzes the description of a user and their profile picture. We used the implementation of the method provided by the authors through a Python library named M3-Inference [55]. The tool outputs scores for three gender categories—male, female, and organization—and four different age ranges.

Before using the detection tool on all the users, to increase its performance, and given the fact that the AN, treatment, and recovered users are not organizations, we defined that only if a user had a score over 0.70, for the organization class, and if this value was higher than the scores for males and females, then this label would be assigned; otherwise, the maximum value among the male and female scores was considered. In addition, if the organization label was assigned to a user, we automatically assigned a specific age group (classified as *an organization*) for all the users classified as organizations. We evaluated the performance of this approach on a group of manually labeled users based on their translated descriptions, where we considered up to 50 users per group. We obtained a macroaverage accuracy of 0.84 for all the gender groups of all the classes and a macroaverage accuracy of 0.80 for all the age groups of all the classes.

##### Visual Aspects

###### Overview

In this section, we describe the use of features extracted from the profile pictures of users and from the images of posts shared by users (RQ1 and RQ7). For the first case, we use pretrained models provided by external sources; for the second case, we train a model on our data set and apply it to the pictures shared by a set of validation users from our groups of interest. We explore visual aspects given that there are physical traits that characterize AN [1], and related work has found visual patterns that characterize similar mental conditions [12,13].

###### Profile Picture

We analyzed 32 features extracted from the pictures of users. We explored the technical features and the detection of emotions and objects. As part of the technical features analyzed, we checked if an image is gray scale, if it is lighter, if it has text, and if it has faces on it. We also analyzed the existence of objects in the pictures. These features are defined through the use of Python libraries such as *imageio* [56] for verifying the brightness of an image, *PIL* [57] for verifying if an image is gray scale, and *the General Recognition AI API* from *Chooch AI* [58], which after taking an image as an input, it outputs the names of elements recognized in the picture, such as texts, clothes, faces, animals, and specific objects. We also detected emotions expressed on the pictures using the Algorithmia facial emotion recognition API, which implements CNN models [59]. The models included in the previous APIs were already trained, so we only ran them over the profile pictures in our data set, and no rights over a further use of these images were granted to the tools’ owners. In addition, none of the images were observed by any human annotator, and only the extracted features were stored.

To analyze the existence of objects in the image and represent a user through these features, we considered a Boolean BoW model. In this model, the names of the objects found were the terms considered, and the value for an object was assigned as 1 if it was found on the picture and 0 if it was not found. Given the sparsity of the model, we only considered objects that appeared in at least five images, which led us to retain 20 features of this type. Regarding the emotions’ features, we assigned to a user (if there are faces on the picture) the emotion with the highest predictive score, and later, to define a score for a group, we considered the ratio of users assigned to a given emotion. The same approach was considered for the technical features and objects detected.

###### Pictures Shared

We explored the pictures shared by users through their individual posts to detect AN-related images. For this purpose, we built two models trained on the images shared by users from three of our groups of interest: AN, focused control, and random control. Two binary classification models were trained: (1) an AN versus focused control image detection model and (2) an AN versus random control image detection model. To train the classifiers, the images of a given user were assigned the same label of the user. The method used to build the model was the one explained in the study by Rodriguez et al [60], which is a method for the inference of personality using the OCEAN model, which refers to the big five personality traits: openness to experience, conscientiousness, extraversion, agreeableness, and neuroticism. Moreover, this method has been previously applied to the detection of suicidal ideation–related images [13]. The resulting model was applied to all the images of a set of users kept for validation purposes, which were not considered in the training process. The output of each model was a score for each class to predict. For a single user, the score corresponding to this feature is given by the average score obtained by the classifier for the AN class over all the user’s images. For the model generation, a total of 64,615 images were used for training and 15,384 for validation. For the first model (AN vs focused control), 278 users were considered for training and 130 for validation purposes. For the second model (AN vs random control), 240 users were considered for training and 106 for validation purposes.

The image classifiers consisted of a CNN [61], and we used residual networks [62], which are popular for improving the back-propagation step in deep architectures. For both classifiers, we considered a model that was initially trained on ImageNet [63] and fine-tuned on the target data set (our training images). As in the study by Ramírez-Cifuentes et al [13], we use a 101-layer ResneXt [64] that uses grouped convolution, an architecture that consists of convolution groups of size 32 with a dimensionality of 8 and a fully connected layer at the end, which is the one that performs the classification between our two defined classes: (1) anorexia related and (2) control image (focused or random control depending on the classifier being trained). The training process was performed for eight epochs with stochastic gradient descent with warm restarts [65]. The learning rate was 0.0001, and the weight decay value was 0.001. We used Nesterov with a momentum of 0.99 on two GTX 1080 Ti graphic cards. We also used dropout (50%) to avoid overfitting.

#### Detection of Precontemplation and Contemplation Phases

According to the labeling approach, within the AN group, we considered users who were probably experiencing AN and did not receive treatment. Among these users, we found those in the precontemplation phase and those at a more advanced stage that might have been willing to start the recovery process (contemplation phase). To detect the elements that distinguish users at the precontemplation stage from those at the contemplation stage, we used a clustering approach. The features selected were those for characterizing users based on their emotions (using the EmoLex features) and that provide an insight on the positivity and negativity of posts as well. We also considered the polarity score, given by the Senti-py library, which classifies a text in the polarity range [0,1]. The use of these features was based on the assumption that people in the precontemplation stage are more positive toward their weight loss and the social support they receive. On the other hand, people in the contemplation phase are at a stage where more negative emotions are manifested due to frustration and lack of control over the weight loss process, which leads to isolation and the affectation of their cognitive capabilities. We used a k-means clustering algorithm for the detection of these communities. Later, we explored the differences among these groups using the values of their features.

## Results

### Comparative Results of Each Perspective

#### Overview

In this section, we present the results obtained for all the features analyzed and explore the differences among the values of these features for each defined group. We also explored certain elements within each perspective, reporting on the topics of interest within each group, the generation of the users’ social network, and the analysis of features generated using classification models.

From a general perspective, the results suggest the relevance of the analysis of the content (texts) generated by users, for which more attributes with significant differences among the groups were identified. This is consistent with the fact that most of the features map aspects considered by psychologists (eg, emotions, risk factors, and the use of vocabulary that indicates harmful behaviors). Regardless of this, the findings on the social network of users also revealed interesting aspects, such as the evident polarization between the focused control users and AN users. We also found a high similarity between the interests of AN users and their followees. In addition, images are relevant for distinguishing AN cases from control cases. Finally, we identified certain elements that distinguish AN users from treatment and fully recovered users.

#### Content Shared and Interests

##### Overview

The results found for this perspective were the most relevant for characterizing AN users (RQ1, RQ3, RQ4, and RQ5). This perspective explored the textual elements from multiple points of view, including linguistic and psychological factors that were particularly useful in distinguishing AN users from control groups. These elements were also important for comparing between our control groups, which were thought to exclusively differ from each other through the use of anorexia-related terms (RQ7). For the majority of the features analyzed for these perspectives, we calculated their median values for each group among all its users. *P* values were also obtained to compare among the following pairs of groups: AN versus treatment, AN versus recovered, AN versus random control, AN versus focused control, and random versus focused control.

##### Linguistic Dimensions

The results for the 24 linguistic dimension features explored are listed in Table 4. We observed many linguistic features that could distinguish AN users from both the control groups. Notably, the use of first-person singular verbs, and consequently first-person singular pronouns, characterized the posts of AN users, along with a high use of negations and a reduced use of articles. In addition, there were more features with highly significant differences between the AN group and the focused control group (features: 22/24, 92%) than between the AN group and the random control group (features: 15/24, 62%). This can be explained by the fact that, as shown in our further analysis, a high percentage of focused control users were organizations (eg, news sites, nutrition, and medical centers), and their linguistic features were quite distinguishable from those of users with personal accounts. This can also be noticed on the elements that distinguish between random and focused control users, as more personal accounts were part of the random control group.

Regarding the differences between the AN group and users in treatment, we observed significant differences in the use of second-person and first-person plural pronouns, which suggests that there might be a change in their attention focus and a higher level of interaction and inclusion with other people. This pattern was even more evident among recovered users.

**Table 4:** Comparative analysis among groups based on linguistic dimensions.

Features	AN^a^, median	Treatment, median	Recovered, median	Random control, median	Focused control, median	AN versus treatment, *P* value^b^	AN versus recovered, *P* value^b^	AN versus random control, *P* value^b^	AN versus focused control, *P* value^b^	Random versus focused control, *P* value^b^
First-person singular verbs	22.2E-03	20.5E-03	17.9E-03	8.14E-03	6.52E-03	.09	<.001^c^	<.001^c^	<.001^c^	.002^d^
First-person plural verbs	9.79E-04	13.8E-04	20.2E-04	17.6E-04	15.5E-04	.01^e^	<.001^c^	<.001^c^	<.001^c^	.03^e^
Second-person singular verbs	3.01E-03	2.90E-03	4.25E-03	2.38E-03	1.99E-03	.34	.008^d^	.001^d^	<.001^c^	.01^e^
Third-person verbs	2.26E-02	2.31E-02	2.50E-02	2.13E-02	1.73E-02	.26	.008^d^	.02^e^	<.001^c^	<.001^c^
Third-person plural verbs	4.99E-03	4.87E-03	5.36E-03	4.45E-03	4.05E-03	.39	.34	.003^d^	<.001^c^	.08
First-person singular pronouns	41.9E-03	41.5E-03	3.0.5E-03	10.1E-03	5.62E-03	.30	<.001^c^	<.001^c^	<.001^c^	<.001^c^
First-person plural pronouns	1.83E-03	2.26E-03	3.75E-03	3.72E-03	3.46E-03	.04^e^	<.001^c^	<.001^c^	<.001^c^	.22
Second-person singular pronouns	9.67E-03	10.6E-03	12.1E-03	8.92E-03	6.52E-03	.16	.003^d^	.24	<.001^c^	<.001^c^
Second-person plural pronouns	2.60E-03	3.38E-03	4.69E-03	2.69E-03	3.12E-03	.004^d^	<.001^c^	.28	<.001^c^	.002^d^
Third-person singular pronouns	3.80E-02	3.93E-02	4.24E-02	4.21E-02	3.91E-02	.05	.005^d^	<.001^c^	.01^e^	.001^d^
Third-person plural pronouns	9.27E-03	9.51E-03	10.6E-03	11.8E-03	10.1E-03	.31	.047^e^	<.001^c^	.06	<.001^c^
Negations	2.66E-02	2.44E-02	2.55E-02	1.98E-02	1.42E-02	.10	.28	<.001^c^	<.001^c^	<.001^c^
Affirmations	7.14E-03	6.46E-03	7.60E-03	7.04E-03	4.50E-03	.33	.24	.19	<.001^c^	<.001^c^
Adverbs	5.24E-02	4.79E-02	4.83E-02	3.81E-02	2.75E-02	.01^e^	.003^d^	<.001^c^	<.001^c^	<.001^c^
Articles	5.67E-02	6.15E-02	6.69E-02	7.06E-02	6.92E-02	.03^e^	<.001^c^	<.001^c^	<.001^c^	.46
Verbs	1.72E-01	1.63E-01	1.71E-01	1.42E-01	1.37E-01	.07	.10	<.001^c^	<.001^c^	.001^d^
Impersonal pronouns	10.9E-02	10.3E-02	10.8E-02	8.28E-02	6.64E-02	.14	.21	<.001^c^	<.001^c^	<.001^c^
Personal pronouns	8.91E-02	9.00E-02	9.00E-02	6.66E-02	5.61E-02	.35	.26	<.001^c^	<.001^c^	<.001^c^
Total pronouns	1.98E-01	1.94E-01	1.94E-01	1.49E-01	1.22E-01	.22	.18	<.001^c^	<.001^c^	<.001^c^
Prepositions	9.81E-02	9.71E-02	10.1E-02	9.97E-02	10.5E-02	.28	.16	.08	<.001^c^	<.001^c^
Past verb tense	1.84E-02	1.88E-02	1.69E-02	1.39E-02	1.18E-02	.43	.11	<.001^c^	<.001^c^	<.001^c^
Present verb tense	1.27E-01	1.22E-01	1.22E-01	1.05E-01	1.00E-01	.13	.17	<.001^c^	<.001^c^	.001^d^
Future verb tense	4.50E-05	7.50E-05	6.70E-05	12.1E-05	12.8E-05	.15	.42	.01^e^	<.001^c^	.07
Median tweet length	14.00	14.00	13.50	12.50	19.00	.45	.43	.001^d^	<.001^c^	<.001^c^

^a^AN: anorexia nervosa.

^b^*P* values were analyzed using Mann-Whitney *U* test.

^c^*P*<.001.

^d^*P*<.01.

^e^*P*<.05.

##### Affective Processes and Emotions

**Figure 2:**
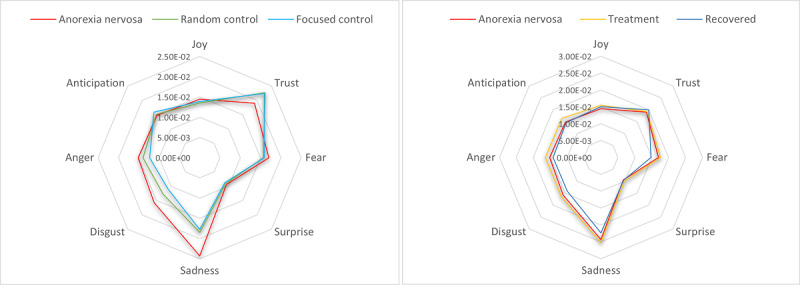
Comparative scores for emotions according to the wheel of emotions by Plutchik. Left: anorexia nervosa and control groups. Right: anorexia nervosa, treatment, and recovered groups.

The results of these features are described in Table 5. As for the linguistic dimensions, there were significant differences between the values of users of the AN and focused control groups. Negative emotions are found mainly for AN and treatment users; this can be observed also on the expression of emotions such as sadness, disgust, and anger, which are significantly higher for AN users than for control users. Within this same comparison, users with AN use more swearing terms and vocabulary that express anxiety and thoughts on their feelings and perceptions. We observe that there are a few attributes with significant differences between AN and treatment users. For joy and positive emotions (LIWC), the scores were significantly higher for treatment users, which might reflect an improvement in the mood of people as they recover from AN. Regarding recovered users, we also observed the existence of less negative emotions and more positive emotions than AN users. In fact, the expressions of anxiety of recovered users were significantly lower than those of AN users. In addition, their high score on social processes and the highly significant values in comparison with AN users suggest an openness to more interactions with other people. Finally, the differences between random and focused control users are mainly observed through the use of swearing terms, the expression of positive emotions, cause and effects, insight, and discrepancies. In this sense, focused control users seem to be more formal and analytic toward things, which meets the characteristics of accounts that represent organizations. For all the groups analyzed, we can observe in Figure 2 a radar chart that expresses the median values for the eight basic emotions defined by the wheel of emotions by Plutchik [66]. We observed the predominance of sadness over all the other emotions in AN users.

**Table 5:** Comparative analysis among groups based on effective processes and emotions.

Features	AN^a^, median	Treatment, median	Recovered, median	Random control, median	Focused control, median	AN versus treatment, *P* value^b^	AN versus recovered, *P* value^b^	AN versus random control, *P* value^b^	AN versus focused control, *P* value^b^	Random versus focused control, *P* value^b^
Swearing	15.4E-03	14.6E-03	12.7E-03	8.06E-03	4.33E-03	.48	.19	<.001^c^	<.001^c^	<.001^c^
Absolutist terms	5.36E-03	5.03E-03	5.09E-03	4.98E-03	3.43E-03	.28	.49	.046^d^	<.001^c^	<.001^c^
Positive emotions (Linguistic Inquiry and Word Count)	5.93E-02	6.23E-02	6.50E-02	6.58E-02	5.79E-02	.04^d^	.03^d^	<.001^c^	.14	<.001^c^
Negative emotions (Linguistic Inquiry and Word Count)	6.88E-02	6.50E-02	6.02E-02	4.97E-02	4.44E-02	.24	.01^d^	<.001^c^	<.001^c^	.01^d^
Anxiety	11.2E-03	12.2E-03	8.91E-03	6.61E-03	6.42E-03	.14	.004^e^	<.001^c^	<.001^c^	.36
Cognitive processes	2.73E-01	2.62E-01	2.64E-01	2.23E-01	2.19E-01	.09	.25	<.001^c^	<.001^c^	.25
Cause and effect	1.79E-02	1.72E-02	1.44E-02	1.35E-02	1.62E-02	.13	.03^d^	<.001^c^	.11	<.001^c^
Insight	3.89E-02	3.73E-02	3.72E-02	3.16E-02	3.60E-02	.30	.21	<.001^c^	.003^e^	<.001^c^
Discrepancies	3.99E-02	3.72E-02	3.94E-02	3.03E-02	2.54E-02	.04^d^	.18	<.001^c^	<.001^c^	<.001^c^
Tentative	4.42E-02	4.53E-02	4.33E-02	3.54E-02	3.49E-02	.20	.46	<.001^c^	<.001^c^	.29
Certainty	1.79E-02	1.74E-02	1.99E-02	1.90E-02	1.56E-02	.38	.05	.27	<.001^c^	<.001^c^
Senses and perceptions	5.03E-02	5.01E-02	4.89E-02	3.70E-02	3.80E-02	.48	.24	<.001^c^	<.001^c^	.20
See	1.23E-02	1.19E-02	1.25E-02	1.06E-02	1.11E-02	.30	.16	.02^d^	.06	.19
Listen	10.6E-03	11.5E-03	13.4E-03	8.87E-03	7.19E-03	.04^d^	.002^e^	<.001^c^	<.001^c^	.03^d^
Feel	16.0E-03	17.0E-03	13.7E-03	8.80E-03	9.30E-03	.42	.01^d^	<.001^c^	<.001^c^	.28
Social processes	1.21E-01	1.21E-01	1.40E-01	1.23E-01	1.14E-01	.33	<.001^c^	.05	.18	.01^d^
References to friends	5.50E-03	4.39E-03	8.09E-03	5.06E-03	4.69E-03	.10	.02^d^	.15	.003^e^	.04^d^
References to family	7.97E-03	7.61E-03	9.57E-03	7.93E-03	7.25E-03	.36	.05	.42	.02^d^	.046^d^
Joy	1.44E-02	1.55E-02	1.50E-02	1.35E-02	1.38E-02	.02^d^	.29	.04^d^	.02^d^	.42
Trust	1.90E-02	1.92E-02	1.99E-02	2.27E-02	2.25E-02	.09	.09	<.001^c^	<.001^c^	.42
Fear	1.70E-02	1.76E-02	1.48E-02	1.57E-02	1.59E-02	.25	.07	.008^e^	.02^d^	.25
Surprise	9.36E-03	9.90E-03	9.50E-03	9.07E-03	8.75E-03	.33	.49	.06	.003^e^	.24
Sadness	2.43E-02	2.53E-02	2.24E-02	1.85E-02	1.78E-02	.30	.02^d^	<.001^c^	<.001^c^	.24
Disgust	1.58E-02	1.65E-02	1.41E-02	1.27E-02	1.10E-02	.48	.08	<.001^c^	<.001^c^	.003^e^
Anger	1.51E-02	1.64E-02	1.42E-02	1.39E-02	1.23E-02	.11	.15	.004^e^	<.001^c^	.02^d^
Anticipation	1.48E-02	1.63E-02	1.46E-02	1.51E-02	1.59E-02	.03^d^	.50	.48	.01^d^	.02^d^
Polarity median score	1.86E-01	1.77E-01	1.87E-01	1.94E-01	1.74E-01	.39	.49	.33	.05	.03^d^
Positive emotions EmoLex	3.60E-02	3.73E-02	3.62E-02	3.82E-02	3.97E-02	.07	.32	.046^d^	<.001^c^	.03^d^
Negative emotions EmoLex	3.60E-02	3.72E-02	3.37E-02	3.34E-02	3.02E-02	.42	.07	<.001^c^	<.001^c^	.03^d^

^a^AN: anorexia nervosa.

^b^*P* values were analyzed using Mann-Whitney *U* test.

^c^*P*<.001.

^d^*P*<.05.

^e^*P*<.01.

##### Personal Concerns and Biological Processes

The results obtained for these features are listed in Table 6. We observe that most of these features are relevant for distinguishing control users from AN users. Control users discuss more about common concerns such as work, leisure, achievement, money, and religion, whereas AN users are more interested in aspects related to their image, which can be seen by their scores on the body, ingest, health, and biological process categories. There was also a significantly higher interest in death, compared with all other categories. For the treatment group, we observed significantly lower values for the ingest and biological process categories, which might be a sign of improvement in their condition compared with AN users. This is more evident in the comparison of AN and recovered users, where there are very significant differences among the same features. Note that the reference to religious aspects is lower for the AN, treatment, and recovered users in comparison with random control users. Regarding random and focused control users, there are differences in the scores for the body, ingest, health, and biological process categories, as these are the ones that refer to signs of the illness. Focused control users are characterized by their use of AN-related terms, and these findings suggest that among the focused control users, we can find people and organizations that often address the topic of AN. Among these, we can find foundations, medical centers, nutritionists, and psychologists. We later validated this assumption through a social network analysis.

**Table 6:** Comparative analysis among groups based on personal concerns and biological processes.

Features	AN^a^, median	Treatment, median	Recovered, median	Random control, median	Focused control, median	AN versus treatment, *P* value^b^	AN versus recovered, *P* value^b^	AN versus random control, *P* value^b^	AN versus focused control, *P* value^b^	Random versus focused control, *P* value^b^
Work	3.21E-02	3.50E-02	3.64E-02	4.95E-02	5.25E-02	.05	.01^c^	<.001^d^	<.001^d^	.09
Achievement	3.87E-02	4.08E-02	3.92E-02	4.39E-02	4.30E-02	.07	.48	<.001^d^	<.001^d^	.18
Leisure	1.70e-02	1.91e-02	1.67e-02	1.99e-02	2.12e-02	.16	.40	<.001^d^	<.001^d^	.27
Home	5.52e-03	5.26e-03	6.38e-03	5.20e-03	5.43e-03	.28	.19	.02^c^	.37	.02^c^
Money	8.22E-03	9.42E-03	11.7E-03	13.8E-03	12.2E-03	.03^c^	.006^e^	<.001^d^	<.001^d^	.003^e^
Religion	2.16E-03	2.67E-03	3.42E-03	5.17E-03	3.38E-03	.10	<.001^d^	<.001^d^	<.001^d^	<.001^d^
Sexual	10.6E-03	11.6E-03	15.6E-03	7.98E-03	7.70E-03	.39	.02^c^	.001^e^	<.001^d^	.22
Death	10.5E-03	8.33E-03	6.50E-03	5.87E-03	6.24E-03	.047^c^	<.001^d^	<.001^d^	<.001^d^	.25
Biological processes	9.02E-02	6.86E-02	6.41E-02	3.21E-02	5.01E-02	.04^c^	.003^e^	<.001^d^	<.001^d^	<.001^d^
Body	2.96E-02	2.45E-02	1.92E-02	1.21E-02	1.55E-02	.07	<.001^d^	<.001^d^	<.001^d^	<.001^d^
Ingest	33.8E-03	17.4E-03	15.3E-03	8.52E-03	11.2E-03	.01^c^	.001^e^	<.001^d^	<.001^d^	<.001^d^
Health	17.7E-03	17.5E-03	17.6E-03	6.83E-03	13.2E-03	.50	.47	<.001^d^	<.001^d^	<.001^d^

^a^AN: anorexia nervosa.

^b^*P* values were analyzed using Mann-Whitney *U* test.

^c^*P*<.05.

^d^*P*<.001.

^e^*P*<.01.

##### Risk Factors Vocabulary

For the use of vocabulary related to risk factors, we noticed that a large number of features were highly significant for the comparison of the AN and control groups. In fact, all the risk factors considered were significant for distinguishing AN from random control users, as shown in Table 7. The use of suicide-related terms is higher for AN users than for all the other groups. Hate and self-loathing terms are found in a lower percentage for recovered users than for AN users and treatment users. We observe that the use of terms related to bullying is higher for treatment and recovered users, which can be explained by the fact that while being on treatment and after recovery, patients are more likely to recognize the issues behind their ED. In general, the scores obtained by all the groups for these features are very low, as these are issues that do not seem to be openly addressed often.

**Table 7:** Comparative analysis among groups based on vocabulary related to risk factors.

Features	AN^a^, median	Treatment, median	Recovered, median	Random control, median	Focused control, median	AN versus treatment, *P* value^b^	AN versus recovered, *P* value^b^	AN versus random control, *P* value^b^	AN versus focused control, *P* value^b^	Random versus focused control, *P* value^b^
Hate	98.4E-05	68.5E-05	33.1E-05	7.60E-05	2.90E-05	.15	<.001^c^	<.001^c^	<.001^c^	.03^d^
Suicide-related terms	4.20E-05	2.20E-05	0	0	0	.04^e^	.002^e^	<.001^c^	<.001^c^	<.001^c^
Self-harm	1.60E-05	4.60E-05	1.00E-05	0	0	.03^e^	.21	<.001^c^	<.001^c^	.02^e^
Work or school problems	8.80E-05	12.0E-05	6.80E-05	1.60E-05	3.10E-05	.41	.16	<.001^c^	<.001^c^	.12
Self-loathing	4.20E-05	1.90E-05	0	0	0	.25	.003^d^	<.001^c^	<.001^c^	.007^d^
Bullying	0	3.00E-06	11.0E-06	0	0	.06	.03^e^	<.001^c^	.02^e^	<.001^c^
Drugs or alcohol abuse	125E-06	143E-06	77.0E-06	6.00E-06	124E-06	.10	.30	<.001^c^	.40	<.001^c^
Lack of social support	0	2.00E-06	0	0	0	.25	.29	<.001^c^	<.001^c^	.01^e^
Relationship issues	5.80E-05	7.00E-05	7.30E-05	0	1.50E-05	.14	.33	<.001^c^	<.001^c^	.001^d^
Use of antidepressants	0	0	0	0	0	.36	.34	<.001^c^	<.001^c^	<.001^c^

^a^AN: anorexia nervosa.

^b^*P* values were analyzed using Mann-Whitney *U* test.

^c^*P*<.001.

^d^*P*<.05.

^e^*P*<.01.

##### Anorexia-Related Vocabulary

These features address the use of vocabulary that describes certain signs and symptoms of AN. The results are presented in Table 8. All the features are highly significant for distinguishing AN users from control cases, and they are all highly significant for distinguishing random from focused control cases. We observed that the scores obtained for the focused control cases were higher than the scores obtained for the random control users. This also happens for the case where recovered and AN users are compared; these users highly differ in the use of vocabulary dedicated to the promotion of AN and vocabulary that expresses concerns regarding body image, body weight, compensatory behavior, and laxatives references, along with caloric restrictions. AN users showed higher scores on these aspects. We also observed that users in the treatment group had lower median values for almost all the features considered, with significant differences in up to four features in comparison with the AN group.

**Table 8:** Comparative analysis among groups based on anorexia-related vocabulary.

Features	AN^a^, median	Treatment, median	Recovered, median	Random control, median	Focused control, median	AN versus treatment, *P* value^b^	AN versus recovered, *P* value^b^	AN versus random control, *P* value^b^	AN versus focused control, *P* value^b^	Random versus focused control, *P* value^b^
Anorexia promotion	35.4E-04	23.5E-04	13.0E-04	4.99E-04	8.47E-04	.02^c^	<.001^d^	<.001^d^	<.001^d^	<.001^d^
Body image	23.5E-04	7.01E-04	4.26E-04	0	1.60E-04	.01^c^	<.001^d^	<.001^d^	<.001^d^	<.001^d^
Body weight	75.7E-05	32.9E-05	9.00E-05	0	8.90E-05	.11	<.001^d^	<.001^d^	<.001^d^	<.001^d^
Food and meals	29.5E-04	21.5E-04	16.8E-04	1.76E-04	5.20E-04	.16	.02^c^	<.001^d^	<.001^d^	<.001^d^
“Eat” as verb	222E-06	100E-06	91.0E-06	0	9.00E-06	.01^c^	.001^e^	<.001^d^	<.001^d^	<.001^d^
Caloric restriction	443E-06	34.0E-06	2.00E-06	0	0	.001^e^	<.001^d^	<.001^d^	<.001^d^	<.001^d^
Binge eating	3.10E-05	3.40E-05	0	0	0	.40	.004^e^	<.001^d^	<.001^d^	<.001^d^
Compensatory behavior and laxatives	9.00E-04	4.88E-04	2.71E-04	0	0	.09	<.001^d^	<.001^d^	<.001^d^	<.001^d^
Exercise	164E-05	91.8E-05	45.2E-05	7.30E-05	43.7E-05	.05	.001^e^	<.001^d^	<.001^d^	<.001^d^

^a^AN: anorexia nervosa.

^b^*P* values were analyzed using Mann-Whitney *U* test.

^c^*P*<.05.

^d^*P*<.001.

^e^*P*<.01.

##### Topics of Interest

In this section, we present the results for the exploration of the topics of interest of users that make use of anorexia-related terms (RQ3), which include AN, treatment, recovered, and focused control users. We assume that the interests of random control users are different and depend on the user. This is due to the fact that we do not consider common interest for these users during the data collection process.

Table 9 shows the top 20 topics of interest for the groups according to the Empath categories. We observe that, apart from the elements in common among groups, only users of the AN group refer to topics such as pain, eating, violence, and suffering. Treatment users have many interests in common with AN users, but we can also observe other topics of interest such as reading, music, and sports. Similarly, recovered users rank topics such as sports and weddings in their list. Focused control users also express interest on different topics, with the highest scored topics being health, communication, business, work, internet, and sports, which matches with our prior assumptions regarding this group. Note that for visualization purposes, the actual median values were multiplied by 1000.

**Figure 3:**
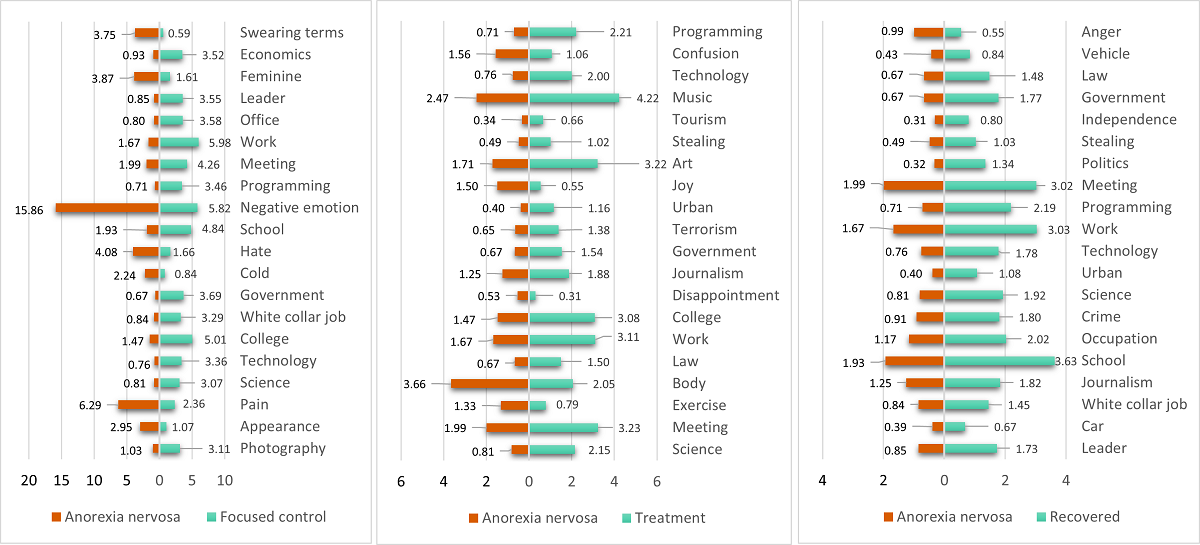
Top 20 topics with most significantly different values (*P*<.05) between the anorexia nervosa group and the focused control, treatment, and recovered groups. The median values for each feature are shown.

To explore the topics of interest in which the groups differed the most from the AN group, we performed the Mann-Whitney *U* test. Figure 3 shows the top 20 topics with the most significantly different values (*P*<.05) between the AN group and the focused control, treatment, and recovered groups. We observe that swearing terms, feminine terms, hate, pain, and appearance obtained high scores for AN users, whereas topics such as economics, college, photography, and work obtained high scores for focused control users. We also observed a limited interest in topics such as music and art for AN users, in comparison with users in treatment, whereas these users (treatment) are less concerned about body and exercise in comparison with users from the AN group. Recovered users are also more concerned about general topics such as law, crime, and politics in comparison with AN users. We report on the percentage of topics found with significant differences among the values for each group (*P*<.05): AN versus focused control, 61% (122/200); AN versus recovered, 40% (80/200); and AN versus treatment, 46.5% (93/200). We also calculated the values for Spearman rank correlation coefficient based on the median values obtained for each topic in each group. The following pairs of groups were compared: AN versus recovered (*ρ*=0.87), AN versus treatment (*ρ*=0.87), AN versus focused control (*ρ*=0.67), treatment versus focused control (*ρ*=0.87), recovered versus focused control (*ρ*=0.87), and treatment versus recovered (*ρ*=0.97). We observe that AN and focused control users are less interested in similar topics, whereas treatment and recovered users’ interests are more correlated with those of the focused control group.

**Table 9:** Top 20 topics of interest (using Empath) among groups that use anorexia-related vocabulary and their median values.

Groups and topics			Value, median
**Anorexia nervosa**			
	Negative emotion	15.86	
	Friends	8.24	
	Speaking	7.67	
	Positive emotion	7.27	
	Children	6.41	
	Pain	6.29	
	Eating	6.19	
	Communication	6.13	
	Optimism	5.93	
	Family	5.91	
	Love	5.60	
	Shame	5.40	
	Violence	5.21	
	Party	4.84	
	Social media	4.70	
	Suffering	4.25	
	Home	4.24	
	Hate	4.08	
	Childish	4.06	
	Feminine	3.87	
**Treatment**			
	Negative emotion	10.50	
	Friends	7.28	
	Positive emotion	6.95	
	Speaking	6.74	
	Social media	6.62	
	Children	6.55	
	Communication	6.06	
	Optimism	5.61	
	Family	5.23	
	Party	4.85	
	Love	4.80	
	Reading	4.51	
	Music	4.22	
	Home	4.10	
	Internet	4.09	
	Musical	3.99	
	Listen	3.94	
	Wedding	3.79	
	Violence	3.63	
	Sports	3.62	
**Recovered**			
	Negative emotion	10.85	
	Friends	9.78	
	Speaking	8.09	
	Positive emotion	7.60	
	Communication	7.01	
	Children	6.66	
	Family	6.46	
	Social media	5.62	
	Home	4.95	
	Party	4.95	
	Optimism	4.91	
	Love	4.91	
	Eating	4.31	
	Wedding	3.95	
	Sports	3.94	
	Giving	3.94	
	Violence	3.89	
	Childish	3.75	
	Pain	3.75	
	Affection	3.71	
**Focused control**			
	Health	9.39	
	Communication	7.41	
	Business	6.90	
	Work	5.98	
	Positive emotion	5.88	
	Internet	5.83	
	Negative emotion	5.82	
	Social media	5.71	
	Speaking	5.54	
	Sports	5.44	
	Messaging	5.03	
	College	5.01	
	Eating	4.92	
	Children	4.86	
	School	4.84	
	Family	4.59	
	Reading	4.41	
	Party	4.30	
	Optimism	4.28	
	Meeting	4.26	

##### Proportion of AN-Related Tweets

Regarding RQ5, the results obtained by the LR classifier at the training approach (cross validation) were as follows: F1 score=0.97, precision=0.98, and recall=0.97, whereas the results for the deep learning approach averaged after multiple runs over the test set were as follows: precision=0.98, recall=0.98, and F1 score=0.98. As the second model obtained slightly better results, it was applied to all the tweets of all the users regardless of the group they belonged to. For each user, the value considered as a feature was the median score obtained by the classifier on all tweets. We then compared the median values of each group analyzed.

We used the Mann-Whitney *U* test to perform an analysis of the median score provided by the classifier to all the users’ tweets. We applied the classifier to all groups of users. The median values for each group are the following: AN (0.23), treatment (0.13), recovered (0.08), random control (0.03), and focused control (0.05). The *P* values for the group comparisons are the following: AN versus recovered (*P*<.001), AN versus treatment (*P*=.004), AN versus focused control (*P*<.001), treatment versus focused control (*P*<.001), and focused control versus random control (*P*=.02). We noticed very significant differences between the AN group and all other groups considered. Notably, the median classifier score obtained by AN users was higher than that obtained by users from all other classes. Moreover, the median values for the groups decreased according to the recovery stage, meaning that the score was lower for recovered users than for treatment users. Note that focused control users obtain a higher score than random control users, as focused control users address AN-related topics.

#### Social Network

##### Overview

Our findings from this perspective (RQ2 and RQ3) provide an insight into the users that are part of the focused control group and verify that organizations and specialists are part of it. We also observed that the structure of the social network could tell us about the different types of accounts that make use of AN-related terms, in particular, through the use of clustering approaches. Our results also showed a high polarization between focused control users and AN users, which was verified by the analysis of the topics of interest between the followees of each user group, where there were significant differences in 75% (150/200) of the analyzed topics.

##### Measures of Interactions and Engagement

The results obtained for these features (Table 10) show that focused control users have a significantly higher median number of followers and followees than AN users. The median number of followers of these users (focused control) shows that these accounts have a higher number of followers than random control users, which might be an indicator of the popularity of these user types that are more likely to be organizations. We also observe that AN users have a reduced number of interactions with other users in comparison with treatment, recovered, and random control users (based on the favorites given). In general, we observe that a reduced number of tweets generated by all user groups are liked or retweeted by other users, probably because they consume this type of information in a discrete way or because they do not generate very popular content.

**Table 10:** Comparative analysis among groups based on interaction and engagement measures.

Features	AN^a^, median	Treatment, median	Recovered, median	Random control, median	Focused control, median	AN versus treatment, *P* value^b^	AN versus recovered, *P* value^b^	AN versus random control, *P* value^b^	AN versus focused control, *P* value^b^	Random versus focused control, *P* value^b^
Number of followers	621.50	815.00	600.00	540.00	1174.00	.02^c^	.26	.26	<.001^d^	<.001^d^
Number of followees	286.50	483.50	289.50	492.00	509.00	.02^c^	.18	<.001^d^	<.001^d^	.24
Given favorites	7746.50	10,893.00	23,955.00	10,085.50	4917.00	.04^c^	.004^e^	.02^c^	.02^c^	<.001^d^
Received favorites	0.00	1.00	0.50	0.00	1.00	.11	.22	.001^e^	.001^e^	<.001^d^
Received retweets	0.00	0.00	0.00	0.00	0.00	.04^c^	.29	.07	<.001^d^	<.001^d^

^a^AN: anorexia nervosa.

^b^*P* values were analyzed using Mann-Whitney *U* test.

^c^*P*<.05.

^d^*P*<.001.

^e^*P*<.01.

##### Analysis of Followees and Community Detection

**Figure 4:**
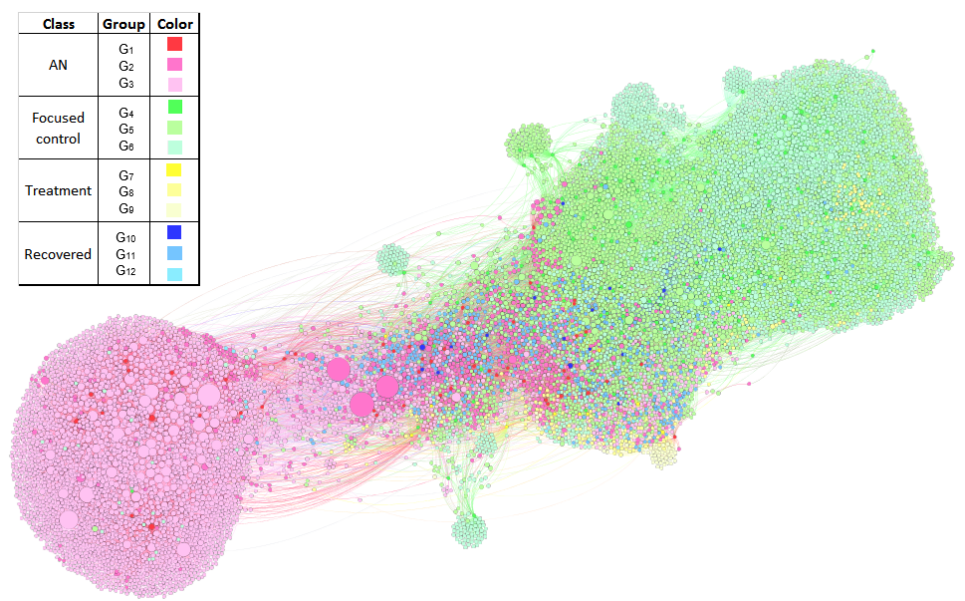
Visualization of the social network of the AN, focused control, treatment, and recovered groups according to the types of users they are mostly followed by. Each group is represented by a different color. Groups associated with the same class have similar colors. AN: anorexia nervosa; G: group ID.

As explained in the *Methods* section, we analyzed the structure of the social network of users using AN-related vocabulary (RQ2). In Table 11, we report on the percentages of nodes belonging to each group defined through the approach previously explained in Table 3. Most of the users considered were part of the focused control group, followed by AN, recovered, and treatment users. For visualization of these groups, we used Gephi, as shown in Figure 4. A total of 99,283 nodes were considered, with each node representing a user. The average number of edges per node (average degree of the graph) was 2.57, the shortest distance between the two most distant nodes in the network (full network diameter) was 15, and the average path length was 4.72, which represents the average number of steps it takes to get from one member of the network to another. The average clustering coefficient was 0.017, which implies that most of the nodes were not related. To ease the visualization and interpretation of the results, we applied a *k*-core filter with *k*=2 to see the maximal subgraph with a minimum degree equivalent to *k*. The number of nodes displayed in Figure 4 is 12,680, and the size of the nodes is given by the page rank score obtained by each node. The graph clearly shows the polarization between the AN and focused control groups, with the few treatment and recovery cases displayed in between and closer to the focused control cases.

**Table 11:** Graph information of subgroups according to the type of followers.

Validation group and subgroup		Nodes user type	Node, %
**AN^a^**			
	G^b^_1_	Labeled as AN	0.05
	G_2_	Mostly followed by G_1_	10.55
	G_3_	Mostly followed by G_2_	12.46
**Focused control**			
	G_4_	Labeled as focused control	0.16
	G_5_	Mostly followed by G_4_	46.56
	G_6_	Mostly followed G_5_	20.57
**Treatment**			
	G_7_	Labeled as treatment	0.01
	G_8_	Mostly followed by G_7_	3.95
	G_9_	Mostly followed G_8_	0.74
**Recovered**			
	G_10_	Labeled as recovered	0.02
	G_11_	Mostly followed by G_10_	4.63
	G_12_	Mostly followed G_11_	0.3

^a^AN: anorexia nervosa.

^b^G: group.

**Figure 5:**
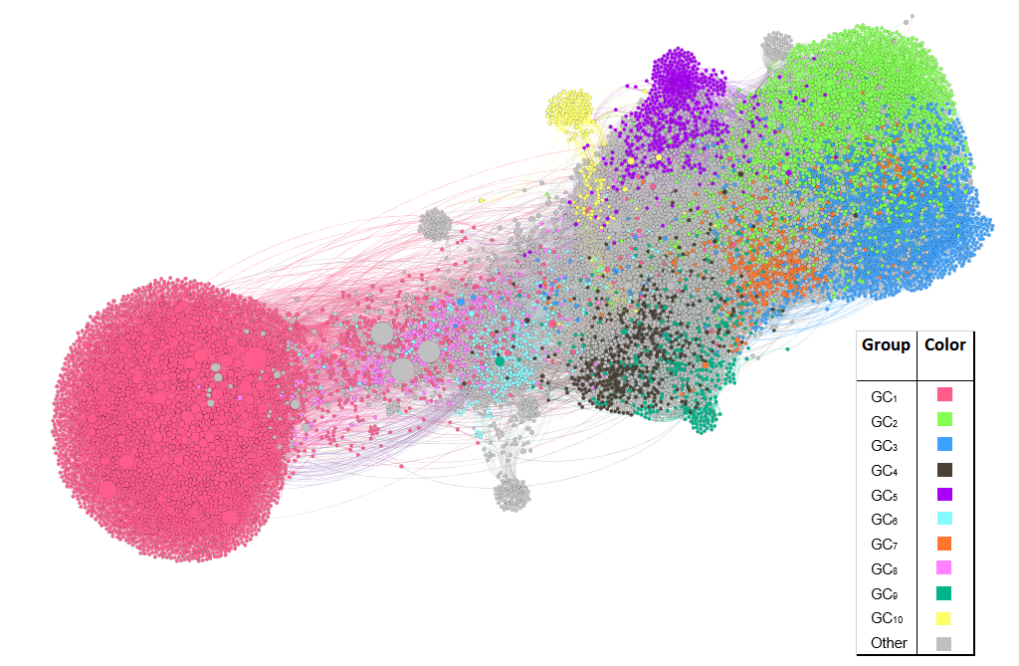
Visualization of the 10 communities with the highest node percentages. Each group corresponds to a community and is represented by a given color. GC: group community.

For further analysis of the full network, we applied a clustering algorithm to detect the communities within it. We found 80 communities and obtained a modularity value of 0.86. As shown in Table 12, we analyzed the descriptions (biographies) of the users of the 10 communities with the highest node percentages. We describe the types of users found in each community and identify the types of users from our annotated groups that are part of each community. Figure 5 shows the network, with the automatically identified communities highlighted. For comparison, we used the same structure displayed in Figure 4. It can be seen that the community with the highest number of nodes is GC_1_, which corresponds to the community of users that are likely to have an ED and users that might be anorexia and bulimia promoters (they correspond to big nodes in the graph, ie, higher page rank, meaning more popular nodes). We also observe two other relevant communities that mainly correspond to focused control cases (GC_2_ and GC_3_) and are characterized by having users that represent organizations and specialists on mental health issues and nutrition centers. We also observe a community that corresponds to news and TV accounts (GC_5_), which also characterizes focused control users. We see that the small number of treatment and recovered users are part of different communities that address multiple topics and that the communities that gather users from different groups are those that involve singers, artists, influencers, and leisure-related topics. These results show that users at the precontemplation and contemplation stages are isolated from accounts that offer assistance to overcome the illness. In this sense, recommender systems might enforce this behavior of the network because they tend to recommend a user to follow similar accounts.

**Table 12:** Description of the types of users identified in each community with the highest node percentages.

Community	Description of the community	Group users identified	Node, %
GC^a^_1_	Users with eating disorders and anorexia and bulimia promoters	Anorexia nervosa	9.65
GC_2_	Organizations, medical centers, and psychologists	Focused control	8.04
GC_3_	Nutritionists and nutrition centers	Focused control	7.37
GC_4_	Varieties: influencers	Focused control	3.88
GC_5_	News and television	Focused control	3.72
GC_6_	Fans of pop singers	AN and recovered	2.69
GC_7_	Undefined varieties	Treatment and focused control	2.54
GC_8_	Undefined varieties	Recovered and AN	2.53
GC_9_	Comics, anime, and drawing	Treatment and focused control	2.31
GC_10_	Uruguayan community	Focused control	2.29

^a^GC: group community identifier.

##### Analysis of Interests Between Users and Their Followees

Regarding the results for the topics of interest of the users who make use of anorexia-related terms (RQ3), Table 13 shows the top 20 topics of interest for our groups’ followees according to the Empath categories. We observe that negative emotions, eating, pain, death, and violence are among the topics most relevant to AN followees. Regarding the other groups, we cannot observe a pattern that would normally characterize each user type; instead, we observe interest in all types of topics, which is more evident in focused control users. We can also observe that topics such as friends, family, children, and parties are relevant for most of the groups.

**Figure 6:**
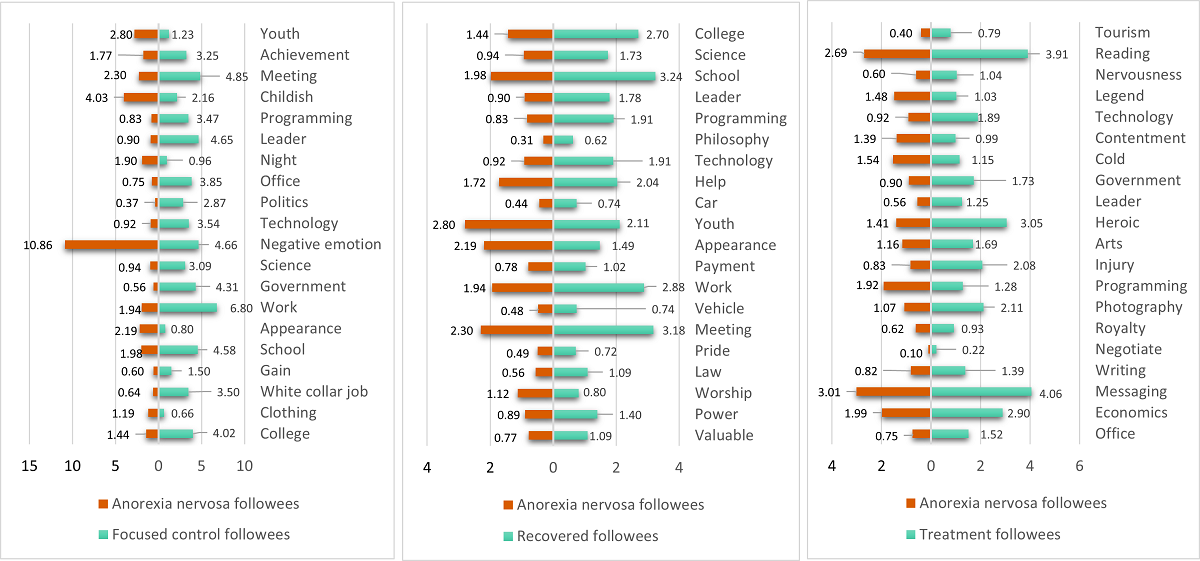
The top 20 topics with most significantly different values (*P*<.05) between the anorexia nervosa followees group and the focused control, recovered, and treatment followees groups. The median values for each feature are shown.

For a better comprehension of the results on this topic analysis task, we explored the topics in which certain followee groups differ the most. We used the Mann-Whitney *U* test for this purpose. Figure 6 shows the top 20 topics, with the most significantly different values (*P*<.05) between the AN followees group and the focused control, treatment, and recovered followee groups. We see a very high value for negative emotion on AN followees in comparison with focused control followees. Appearance is also a topic in which AN followees differ from focused control followees and recovered followees. We also report on the percentage of topics found with significant differences among the median values for the following pairs of groups (*P*<.05): AN followees and recovered followees, 45% (90/200); AN followees and focused control followees, 75% (150/200); AN followees and treatment followees, 48% (96/200); AN and AN followees, 22% (44/200); and recovered and recovered followees, 21% (42/200). From this, we observe that AN users and recovered users differ the least in their interests with their own followees. AN followees and focused control followees show the biggest difference in interests. We also calculated the values for Spearman rank correlation coefficient based on the median values obtained for each topic in each group. The pairs of groups were as follows: AN and AN followees (*ρ*=0.96), treatment and treatment followees (*ρ*=0.97), recovered and recovered followees (*ρ*=0.96), focused control and focused control followees (*ρ*=0.97), AN followees and treatment followees (*ρ*=0.93), AN followees and recovered followees (*ρ*=0.93), AN followees and focused control followees (*ρ*=0.69), treatment followees and focused control followees (*ρ*=0.86), and recovered followees and focused control followees (*ρ*=0.86). From these results, we can say that for all the groups, their interests are highly similar to those of their followees; however, the interests of the treatment and recovered followees groups are more highly correlated to the focused control group followees than the AN followees group, indicating a change in interest through the evolution of the disorder.

**Table 13:** Top 20 topics of interest and their Empath median values for the groups that use anorexia-related vocabulary followees.

Groups and topics			Value, median
**Anorexia nervosa**			
	Negative emotion	10.86	
	Friends	7.58	
	Positive emotion	7.30	
	Speaking	6.50	
	Communication	5.93	
	Optimism	5.85	
	Children	5.80	
	Social media	5.71	
	Party	5.62	
	Love	5.09	
	Family	4.69	
	Childish	4.03	
	Giving	4.00	
	Eating	3.93	
	Home	3.83	
	Pain	3.72	
	Death	3.72	
	Wedding	3.70	
	Violence	3.62	
	Celebration	3.42	
**Treatment**			
	Negative emotion	7.92	
	Friends	7.76	
	Positive emotion	7.02	
	Communication	6.96	
	Social media	6.83	
	Speaking	6.42	
	Children	5.48	
	Party	5.43	
	Optimism	5.22	
	Family	4.82	
	Love	4.28	
	Internet	4.15	
	Music	4.09	
	Messaging	4.06	
	Listen	4.02	
	Musical	3.92	
	Reading	3.91	
	Wedding	3.76	
	Celebration	3.74	
	Childish	3.72	
**Recovered**			
	Negative emotion	9.73	
	Friends	7.71	
	Positive emotion	7.23	
	Communication	7.15	
	Speaking	6.87	
	Social media	5.69	
	Optimism	5.55	
	Party	5.22	
	Children	5.20	
	Family	4.98	
	Internet	4.33	
	Giving	4.00	
	Love	3.96	
	Messaging	3.83	
	Reading	3.73	
	Wedding	3.70	
	Celebration	3.67	
	Listen	3.57	
	Home	3.52	
	Childish	3.47	
**Focused control**			
	Business	7.29	
	Communication	7.04	
	Work	6.80	
	Positive emotion	6.41	
	Internet	5.72	
	Social media	5.36	
	Party	4.86	
	Meeting	4.85	
	Speaking	4.79	
	Negative emotion	4.66	
	Leader	4.65	
	Reading	4.60	
	School	4.58	
	Messaging	4.57	
	Children	4.56	
	Occupation	4.50	
	Family	4.49	
	Optimism	4.35	
	Government	4.31	
	Celebration	4.12	

#### Behavioral Aspects

**Figure 7:**
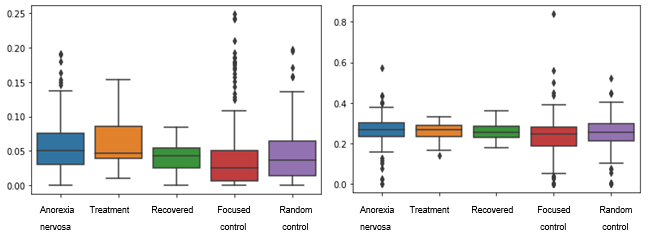
Comparative box plots of the sleep period tweeting ratio (left) and the weekend tweet count ratio (right) of each user group.

The results of the behavioral aspects (RQ1) analyzed (Table 14) showed that AN users tweeted more on weekends compared with control groups. In addition, the median time between tweets was lower for AN users (they tweeted more frequently) in comparison with random and focused control users. We also observed that the tweeting ratio during sleeping periods was significantly higher for AN users than for the control groups. This might indicate some sleep alteration, which is a usual sign in EDs and other associated mental issues, such as depression. Regarding the tweeting periods during the year, we see that between December and February (winter in Europe and summer in most countries of South America) users from the AN group tweeted less than users from all the other groups. However, we cannot match this finding to a clinical fact related to the seasons of the year, given the lack of information regarding the users’ location. Figure 7 shows the comparative box plot of the values for each group on the sleep period tweeting ratio and the weekend tweet count ratio.

**Table 14:** Comparative analysis among groups based on behavioral aspects.

Features	AN^a^, median	Treatment, median	Recovered, median	Random control, median	Focused control, median	AN versus treatment, *P* value^b^	AN versus recovered, *P* value^b^	AN versus random control, *P* value^b^	AN versus focused control, *P* value^b^	Random versus focused control, *P* value^b^
Working week tweet count ratio	0.73	0.73	0.75	0.75	0.75	.37	.15	.03^c^	<.001^d^	.01^c^
Weekend tweet count ratio	0.27	0.27	0.25	0.25	0.25	.37	.15	.03^c^	<.001^d^	.01^c^
Median time between tweets	625.25	701.00	1187.50	4063.75	1088.00	.43	.15	<.001^d^	.005^e^	<.001^d^
Sleep period tweeting ratio	0.05	0.05	0.04	0.04	0.03	.30	.07	<.001^d^	<.001^d^	.001^e^
Awake period tweeting ratio	0.95	0.95	0.96	0.96	0.97	.30	.07	<.001^d^	<.001^d^	.001^e^
Normalized tweet count per year quarter: December to February	0.01	0.16	0.15	0.18	0.16	<.001^d^	.02^c^	<.001^d^	<.001^d^	.01^c^
Normalized tweet count per year quarter: March to May	0.01	0.23	0.21	0.23	0.21	.001^e^	.11	<.001^d^	.01^c^	.001^e^
Normalized tweet count per year quarter: June to August	0.27	0.32	0.27	0.25	0.24	.39	.29	.10	.004^e^	.06
Normalized tweet count per year quarter: September to November	0.36	0.24	0.27	0.28	0.31	.002^e^	.20	.001^e^	.32	.002^e^
Number of tweets created since the account creation	7910.00	21,038.50	18,409.00	23,291.50	21,463.00	.004^e^	.12	<.001^d^	<.001^d^	.49

^a^AN: anorexia nervosa.

^b^*P* values were analyzed using Mann-Whitney *U* test.

^c^*P*<.05.

^d^*P*<.001.

^e^*P*<.01.

#### Demographics

**Figure 8:**
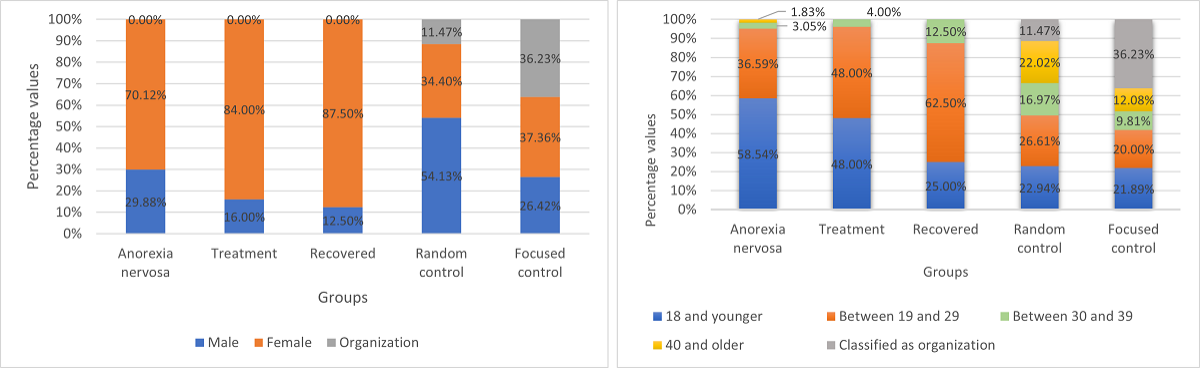
Composition of the anorexia nervosa, treatment, recovered, and control user groups according to gender and age. Each age and gender subgroup is represented by a color.

Using the age and gender inference tool, we obtained the percentages of users corresponding to demographic categories (RQ1) for each group (Figure 8). Most of the AN, treatment, and recovered users were young women. These results are compatible with the statistics that mention that the incidence rates for AN are the highest for women aged 15-19 years [54]. We also observe that a considerable number of users in the focused control group represent organizations. When comparing the ratios of users belonging to each gender per group (Table 15), we see differences between the AN and control groups due to the number of female users. We also observed differences between the focused control and random control groups, as there were fewer organizations in the random control group. Regarding age (Table 16), we observe that AN users differ from the control groups because of the large number of AN users aged ≤18 years. We also find differences between the AN and recovered groups, as recovered users are normally older than AN users. This is consistent with the fact that a full recovery process often takes years, and therefore, users get older as the recovery stages are reached.

**Table 15:** Comparative analysis among groups based on gender.

Gender	AN^a^ versus treatment, *P* value^b^	AN versus recovered, *P* value^b^	AN versus random control, *P* value^b^	AN versus focused control, *P* value^b^	Random versus focused control, *P* value^b^
Male	.15	.14	<.001^c^	.44	<.001^c^
Female	.15	.14	.04^d^	<.001^c^	.50
Organization	N/A^e^	N/A	<.001^c^	<.001^c^	<.001^c^

^a^AN: anorexia nervosa.

^b^*P* values were analyzed using proportions *z* test.

^c^*P*<.001.

^d^*P*<.05.

^e^N/A: not applicable.

**Table 16:** Comparative analysis among groups based on age, including users classified as organizations.

Age groups, years	AN^a^ versus treatment, *P* value^b^	AN versus recovered, *P* value^b^	AN versus random control, *P* value^b^	AN versus focused control, *P* value^b^	Random versus focused control, *P* value^b^
≤18	.32	.01^c^	.002^d^	<.001^e^	.78
19-29	.27	.04^c^	.31	<.001^e^	.09
30-39	.80	.06	<.001^e^	.009^d^	.02^c^
≥40	.50	.59	<.001^e^	<.001^e^	.003^d^

^a^AN: anorexia nervosa.

^b^*P* values were analyzed using proportions *z* test.

^c^*P*<.05.

^d^*P*<.01.

^e^*P*<.001.

#### Visual Aspects

The visual aspects analyzed address the profile picture of the user and the images shared on their posts (RQ1). We found significant differences between the groups’ profile pictures. Regarding the technical aspects (Table 17), we can observe that focused control users are likely to be distinguished from AN users because of the presence of text in their profile pictures. This also applies to random control users, who tend to use text as well but in a lower ratio than focused control users. These findings can be explained by the use of logos in the profile pictures of the accounts of organizations. In addition, AN users’ pictures are significantly darker than focused control users’ pictures. In terms of the emotions detected (Table 18), we observed that treatment users expressed more neutral emotions. We also notice that sadness characterizes AN users, which are the only ones with a ratio of users showing such expressions. On the objects detected (Table 19), there were significant differences in the existence of clothing elements between the AN and control groups, along with the appearance of hands, shorts, and accessories, which might suggest that more full-body pictures are shared by AN users, which might consequently imply a higher interest in their appearance. We also observe that there is a high ratio of posters on the control users’ profiles, which validates our prior assumption about the representation of organizations. We also observed that few men were identified on pictures of users of the AN group, whereas women were identified on more than half of the AN profile’s pictures.

**Table 17:** Comparative analysis among groups based on technical aspects of profile pictures.

Features	AN^a^ ratio of users	Treatment ratio of users	Recovered ratio of users	Random control ratio of users	Focused control ratio of users	AN versus treatment, *P* value^b^	AN versus recovered, *P* value^b^	AN versus random control, *P* value^b^	AN versus focused control, *P* value^b^	Random versus focused control, *P* value^b^
Is gray scale	0.09	0.13	0.17	0.05	0.03	.78	.57	.31	.06	.43
Is lighter	0.50	0.63	0.50	0.42	0.65	.51	.99	.30	.03^c^	<.001^d^
Has text	0.06	0.13	0.00	0.22	0.34	.51	.53	.008^e^	<.001^d^	.045^c^
Has faces	0.28	0.50	0.33	0.25	0.26	.21	.79	.66	.79	.82

^a^AN: anorexia nervosa.

^b^*P* values were analyzed using proportions *z* test.

^c^*P*<.05.

^d^*P*<.001.

^e^*P*<.01.

**Table 18:** Comparative analysis among groups based on emotions detected from profile pictures.

Features	AN^a^ ratio of users	Treatment ratio of users	Recovered ratio of users	Random control ratio of users	Focused control ratio of users	AN versus treatment, *P* value^b^	AN versus recovered, *P* value^b^	AN versus random control, *P* value^b^	AN versus focused control, *P* value^b^	Random versus focused control, *P* value^b^
Neutral	0.00	0.38	0.00	0.05	0.09	<.001^c^	N/A^d^	.06	.02^e^	.34
Sad	0.09	0.00	0.00	0.00	0.00	.37	.43	.002^f^	<.001^c^	N/A
Fear	0.05	0.00	0.17	0.03	0.02	.53	.23	.61	.20	.43
Surprise	0.02	0.00	0.00	0.00	0.01	.72	.76	.22	.61	.39
Angry	0.09	0.13	0.00	0.06	0.07	.78	.43	.46	.56	.83
Happy	0.03	0.00	0.17	0.08	0.09	.61	.12	.18	.12	.80
Disgust	0.00	0.00	0.00	0.03	0.02	N/A	N/A	.15	.32	.43

^a^AN: anorexia nervosa.

^b^*P* values were analyzed using proportions *z* test.

^c^*P*<.001.

^d^N/A: not applicable.

^e^*P*<.05.

^f^*P*<.01.

**Table 19:** Comparative analysis among groups based on objects detected from profile pictures.

Features	AN^a^ ratio of users	Treatment ratio of users	Recovered ratio of users	Random control ratio of users	Focused control ratio of users	AN versus treatment, *P* value^b^	AN versus recovered, *P* value^b^	AN versus random control, *P* value^b^	AN versus focused control, *P* value^b^	Random versus focused control, *P* value^b^
Poster	0.00	0.00	0.00	0.09	0.24	N/A^c^	N/A	.01^d^	<.001^e^	.004^f^
Clothing	0.75	1.00	0.50	0.53	0.33	.11	.19	.005^f^	<.001^e^	.003^f^
Person	0.28	0.13	0.00	0.32	0.12	.34	.13	.58	.007^f^	<.001^e^
Man	0.05	0.00	0.00	0.32	0.16	.53	.59	<.001^e^	.03^d^	.003^f^
Dress	0.03	0.00	0.17	0.01	0.02	.61	.12	.34	.74	.47
Boy	0.02	0.00	0.00	0.02	0.01	.72	.76	.81	.61	.40
Tree	0.02	0.00	0.17	0.05	0.03	.72	.03^d^	.23	.53	.43
Human hand	0.06	0.00	0.00	0.00	0.01	.47	.53	.01^d^	.02^d^	.39
Fashion accessory	0.08	0.00	0.00	0.02	0.02	.41	.48	.08	.03^d^	.77
Flower	0.03	.0.00	0.00	0.01	0.04	.61	.66	.34	.79	.19
Glasses	0.00	0.13	0.00	0.05	0.03	.004^f^	N/A	.06	.16	.43
Animal	0.00	0.00	0.00	0.02	0.02	N/A	N/A	.25	.22	.90
Shorts	0.06	0.13	0.00	0.00	0.00	.51	.53	.01^d^	.004^e^	N/A
Jeans	0.03	0.00	0.00	0.02	0.01	.61	.66	.68	.21	.40
Human eye	0.06	0.00	0.00	0.01	0.01	.47	.53	.06	.02^c^	.83
Cat	0.00	0.13	0.00	0.01	0.02	.004^f^	N/A	.41	.22	.47
Footwear	0.09	0.00	0.00	0.05	0.02	.37	.43	.31	.01^d^	.12
Human nose	0.02	0.00	0.00	0.02	0.00	.72	.76	.81	.16	.10
Girl	0.19	0.25	0.17	0.0	0.03	.67	.90	.006^f^	<.001^e^	.43
Woman	0.58	0.75	0.67	0.18	0.22	.35	.67	<.001^e^	<.001^e^	.38

^a^AN: anorexia nervosa.

^b^*P* values were analyzed using proportions *z* test.

^c^N/A: not applicable.

^d^*P*<.05.

^e^*P*<.001.

^f^*P*<.01.

Finally, when it comes to the exploration of the pictures posted by users, we developed two classifiers: (1) one trained with the images of users from the AN group and users from the focused control group and (2) another trained with the images of users from the AN group and users from the random control group. The results showed that there were highly significant differences between the AN and control groups (*P*<.001 for both comparisons). The median of the aggregated scores of the first classifier for the AN class (AN vs focused control) for a set of 130 validation users was 0.73, whereas the median value for focused control users was 0.36. This means that a higher number of pictures related to AN were found on the posts of AN users. When analyzing the median of the aggregated scores of the second classifier (AN vs random control), on a set of 106 validation users, the median value for AN users was 0.78 and for random control users was 0.54. We observed lower aggregated scores for both control cases, meaning that these users share fewer AN-related pictures. These results show that the pictures are informative for the detection of users with AN.

Regarding the visual content of the images posted by users with AN, there are some visual patterns that emerge after training CNNs, as described previously. These visual patterns were found to be quite characteristic in such posted images because they received the highest scores by the CNN. As expected, most images correspond to body objectification, that is, selfies, extremely thin body parts (mostly legs), and (altered) images of unrealistic ideals of body size. There are also several images of scales showing (rather low) weight numbers. Finally, and less frequently, we can identify rather healthy food images, mostly salads and fruits.

### Detection of Precontemplation and Contemplation Phases

**Figure 9:**
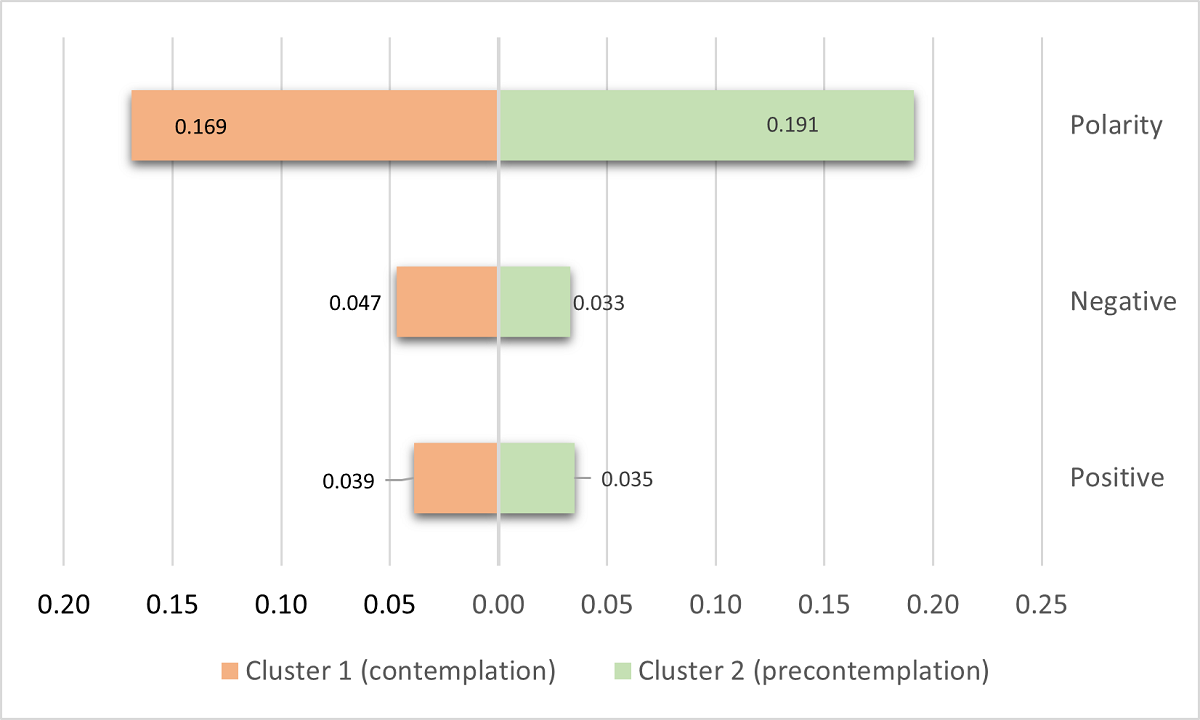
Polarity (calculated using Senti-py) and positivity and negativity (calculated using EmoLex) values for the detected precontemplation and contemplation clusters.

After performing the clustering approach with k-means, we obtained two clusters of users among those labeled as AN (RQ6). We analyzed both groups and found strong indicators of the groups identified as our contemplation (cluster 1) and precontemplation (cluster 2) groups. This was mainly because the precontemplation cluster was characterized by higher values of positivity compared with its negativity, whereas the opposite occurred for the contemplation cluster (Figure 9). In addition, the median polarity score for the precontemplation cluster (0.1915) was significantly higher (*P*<.001) than that for the contemplation (0.1694) cluster. In fact, there were significant differences (*P*<.001) in the values for all features considered in the clustering approach. In addition, the differences between opposite (positive and negative) emotions such as joy and sadness are lower for the values of the precontemplation cluster, as the early stages of the illness are characterized by signs of the person feeling in control of the weight loss process and being enthusiastic about their progress and the social support received. The median values for all features analyzed for each cluster are displayed in Table 20. Among the AN users, a total of 115 users were assigned to the precontemplation cluster, and 56 users were assigned to the contemplation cluster.

**Table 20:** Median values of the features considered for the contemplation and precontemplation clusters.

Emotion	Cluster 1 (contemplation), median	Cluster 2 (precontemplation), median
Joy	15.92	13.85
Sadness	32.88	21.95
Anticipation	17.08	14.13
Surprise	11.49	8.72
Trust	20.36	18.30
Disgust	22.22	13.92
Anger	19.44	13.72
Fear	23.23	14.91

We can observe that the results obtained for this task are consistent with the characteristics of people going through each stage; however, there is no annotated information for the validation of the classification performed, and this is an aspect that needs to be addressed in the future. Ours is the first approach to address this classification task, which is relevant given that users at the contemplation stage are more prone to seek help, and therefore, different ways to approach each type of user may be considered.

## Discussion

### Principal Findings

This study analyzed the elements that characterize Spanish-speaking users with AN during the stages of their recovery process. We have considered multiple points of view, including linguistic, psychological, relational, behavioral, demographical, and visual aspects, for our analysis. We believe that this is the first approach to analyze both users in treatment and those fully recovered in multiple perspectives. Furthermore, we identified the topics of interest of different types of users that used AN-related vocabulary, along with those of their followees. In this section, we report the main findings according to the RQs addressed.

With regard to RQ1, we found multiple elements that characterize and distinguish users with AN at the early, treatment, and full recovery stages. AN users tweeted more frequently at night and on weekends than focused control users. These results are consistent with clinical findings that suggest that patients with AN often report poor sleep quality and reduced sleep time [67]. In addition, the image results indicate that the analysis of visual elements is relevant for the detection of AN cases and focused control cases. In particular, our findings showed that the features extracted from the content generated by users are the most relevant for characterizing AN users, especially those related to linguistic and psychological factors, including terms that describe risk factors and the signs and symptoms of AN (ie, anorexia-related vocabulary).

With regard to RQ4, we determined the linguistic attributes that characterize Spanish-speaking users with AN and found that similar to a related work on English texts [6], the high use of first-person singular pronouns and verbs conjugated with these pronouns distinguishes AN users from control and recovered cases (*P*<.001). We have also observed that the AN group is characterized by a significantly lower use of articles and higher use of impersonal pronouns than control users (*P*<.001), which, to the best of our knowledge, has not been reported in any related work in English.

Our findings also reinforced the relevance of textual elements through the development of a deep learning model for the detection of AN-related tweets, which was constructed to address RQ5. We explored the change in the ratio of posts related to AN tweeted by users at each stage and found that users at the early stages of AN posted more AN-related tweets. In addition, highly significant differences among AN cases, recovered cases, and control cases (*P*<.001) were observed. There were also very significant differences between the AN and treatment groups (*P*=.004) in terms of this feature, that is, the proportion of tweets related to AN significantly changed depending on the recovery stage, indicating the progress in the recovery process of social media users with AN.

Among the relational factors explored (RQ2), we found that the AN and focused control groups could be identified by analyzing the structure of their social network (clustering approach). Among focused control users, there were several organizations and specialists for the treatment and prevention of EDs. The high polarization noted among the AN- and focused control–related communities reinforces the findings of previous studies conducted on networks of English speakers [17,18], which reported limited interactions between pro-ED and prorecovery communities (RQ4). From a psychological perspective, these findings can be explained by the elements that characterize people at the precontemplation stage according to the transtheoretical model of health behavior change, where people are in denial of their unhealthy conditions and tend to feel supported by their equals (pro-ED community members) [4,5], resulting in a rejection of prorecovery content. Regarding the topics of interest of users and their followees (RQ3), we found that the interests of AN users and their followees were highly correlated (*ρ*=0.96). We also observed a higher correlation between the treatment followees and focused control followee groups (*ρ*=0.86) and the recovered followees versus focused control followees groups (*ρ*=0.86) in comparison with the AN followees versus focused control followees groups (*ρ*=0.69). These results suggest that more interests are shared with focused control users as the recovery process advances.

With regard to RQ6, we posed a question regarding the detection of users at the precontemplation stage and users at the contemplation stage. After applying a clustering process and using emotion indicators, we identified two clusters, where one was characterized by a predominance of positive emotions over the negative ones, suggesting that users at the precontemplation stage could be the members of this cluster. On the other hand, the second cluster showed values that characterize people at the contemplation stage, where negative emotions predominate.

Finally, to address RQ7, where we explore the existence of differences among our control groups (random and focused control users), we observed that the focused control group had three times more organizations’ accounts than the random control group. We also noticed that the main differences among these groups were found in linguistic attributes, especially for focused control users that were characterized by the use of a reduced number of swearing terms and more anorexia-related vocabulary terms. These findings complement our prior assumptions that focused control users were mostly organizations, specialists, and clinicians, corresponding to a prorecovery community [6,17,18].

Given our findings, another relevant aspect to discuss is the indirect and unintended role of social platforms in the promotion of harmful content among users with and without EDs. A previous study [68] reported significant decreases in caloric intake among people exposed to pro-ED websites between preexposure and postexposure. As recommender systems are designed, users with AN are likely to be recommended to follow users similar to them (other AN users), and we believe that this indirectly contributes to the reinforcement of unhealthy habits. Tools that are aware of these risks must thus be developed.

### Limitations

The analyses performed in this study present certain limitations that are mainly given by the structure of the social platform analyzed, which does not provide explicit information regarding elements relevant to our analysis, such as the location, age, gender, or medical records of users. This is the main reason that has led us to infer information based on the analysis of the users’ posts; therefore, the accuracy of our results is limited to the performance of the tools we have applied. This applies to the demographical features inferred (age groups and gender); the analysis of elements that are related to the signs and symptoms of anorexia (terms related to risk factors and AN-related vocabulary); image analysis tools; and the inference of aspects that involve the location of the user, such as the weekend tweet count ratio and the sleep period tweeting ratio. This last feature is calculated in a way that overcomes the issue of not knowing the difference in the posting time according to the user’s time zone. This aspect is also an issue regarding the tweeting frequency in different periods of the year, as the seasons change according to the location of users. In this sense, a Spanish variant classifier could be useful to distinguish users from different locations; however, this would still be insufficient for the cases of migrants.

We did not measure the biases introduced by the platform in our data set; however, our samples seemed to be representative of the reality in terms of age and gender despite most Twitter users being male and middle-aged [69]. The AN group users were mostly female teenagers, consistent with the age group with the highest incidence rate (15- to 19-year-old females) for AN [54]. We also considered the limitations owing to the accuracy of the translation of terms to English for the annotation and use of Empath as a topic detection tool.

We also considered the limitations that pose the characteristics of users who have a preference for Twitter as a platform and who choose to make their tweets publicly available, which might differ from those that keep their profiles private. It is important to recall that our study is limited to users who make use of Twitter; therefore, the analysis of the behavior of users from other social platforms and even of people with AN that do not have accounts on any social platforms is out of our reach.

### Reproducibility and Ethical Concerns

This work was approved by the ethical review board of Pompeu Fabra University. To avoid processing and storing personal or sensitive data, a proper process of data transformation and anonymization was followed. We only stored the extracted transformed features. Our approach corresponded to an observational study, with findings that implied that the detection of AN on social media is viable; however, the further development and deployment of detection tools are required. Thus, it is necessary to perform a prior and proper risk-benefit assessment accompanied by the analysis of the legal frameworks that regulate such activities. It is also important to address the potential misusage capabilities of such tools [16,70].

Regarding the reproducibility of this work, Twitter’s policies on the distribution of the data collected through its API should be respected. No information that could lead to the identification of the users included in our study will be shared, as we did not store any personal information [71]. However, the values of the features calculated are available upon reasonable request and after a proper evaluation of the use purpose.

### Future Work

We believe that our findings are relevant to the development of predictive models that can assist specialists in the detection of users with AN and that can display indicators for risk factors as well as signs and symptoms that characterize AN. A proper risk assessment process should be performed before the deployment of such models.

Another aspect that we intend to analyze is the area of recommender systems. According to our findings, AN users mainly followed AN-related content, which reinforces their harmful behaviors. Thus, a recommender system that breaks this bubble and reduces the polarization between AN and focused control users can be an interesting path to follow. This is relevant considering that most recommender systems of social platforms are focused on matching users with shared interests, which in this case, might not be appropriate.

## Abbreviations

AN: anorexia nervosa
API: application programming interface
BoW: bag-of-words
CNN: convolutional neural network
DTTR: day time tweets ratio
ED: eating disorder
LIWC: Linguistic Inquiry and Word Count
LR: logistic regression
pro-ED: pro-eating disorder
RQ: research question
STTR: sleep time tweets ratio
