# Correction: Characterization of Anorexia Nervosa on Social Media: Textual, Visual, Relational, Behavioral, and Demographical Analysis

**DOI:** 10.2196/33447

**Published:** 2021-10-12

**Authors:** Diana Ramírez-Cifuentes, Ana Freire, Ricardo Baeza-Yates, Nadia Sanz Lamora, Aida Álvarez, Alexandre González-Rodríguez, Meritxell Lozano Rochel, Roger Llobet Vives, Diego Alejandro Velazquez, Josep Maria Gonfaus, Jordi Gonzàlez

**Affiliations:** 1 Department of Information and Communication Technologies Universitat Pompeu Fabra Barcelona Spain; 2 UPF Barcelona School of Management Barcelona Spain; 3 Institute for Experiential AI Northeastern University Boston, MA United States; 4 Department of Mental Health Centro de Investigación Biomédica en Red de Salud Mental Parc Tauli University Hospital Sabadell Spain; 5 Parc Tauli Research and Innovation Institute Sabadell Spain; 6 Autonomous University of Barcelona Bellaterra Spain; 7 Fundación Instituto de Trastornos Alimentarios Barcelona Spain; 8 Computer Vision Center Universitat Autonoma de Barcelona Bellaterra Spain; 9 Visual Tagging Services Bellaterra Spain

In “Characterization of Anorexia Nervosa on Social Media: Textual, Visual, Relational, Behavioral, and Demographical Analysis” (J Med Internet Res 2021; 23(7):e25925), the authors noted one error.

In the originally published paper, a reference was mistakenly placed in the following sentence in the Introduction – Background section:

EDs, such as AN, are strongly related to risk factors including perfectionistic traits, parenting style [2], and the existence of comorbid mood disorders such as depression, of which 33%-50% of anorexia patients experience.

To address this issue and to avoid a misinterpretation of the conclusions of Reference 2, which analyses but does not state that there is a strong relation between AN and parenting style, the sentence has been corrected as follows:

EDs, such as AN, are related to risk factors including perfectionistic traits [2], and the existence of comorbid mood disorders such as depression, of which 33%-50% of anorexia patients experience.

The correction will appear in the online version of the paper on the JMIR Publications website on October 12, 2021, together with the publication of this correction notice. Because this was made after submission to PubMed, PubMed Central, and other full-text repositories, the corrected article has also been resubmitted to those repositories.

